# Taxonomic contributions to Pleosporales and Kirschsteiniotheliales from the Xizang Autonomous Region, China

**DOI:** 10.1080/21501203.2025.2493072

**Published:** 2025-05-27

**Authors:** Shu-Cheng He, Kevin D. Hyde, Ruvishika S. Jayawardena, Vinodhini Thiyagaraja, Dhanushka N. Wanasinghe, Yun-Wei Zhao, Zhi-Yang Wang, Tong Cai, Yan-Yan Yang, F. Al-Otibi, Zhuliang Yang, Qi Zhao

**Affiliations:** a State Key Laboratory of Phytochemistry and Natural Medicines, Kunming Institute of Botany, Chinese Academy of Sciences, Kunming, China; b Center of Excellence in Fungal Research, Mae Fah Luang University, Chiang Rai, Thailand; c Department of Botany and Microbiology, College of Science, King Saud University, Riyadh, Saudi Arabia; d Department of Soil Science, College of Food and Agriculture Sciences, King Saud University, Riyadh, Saudi Arabia; e Honghe Center for Mountain Futures, Kunming Institute of Botany, Chinese Academy of Sciences, Honghe County, China; f State Key Laboratory for Conservation and Utilization of Bio-Resources in Yunnan, Yunnan University, Kunming, China; g Faculty of Agriculture, Chiang Mai University, Chiang Mai, Thailand; h Key Laboratory of Forest Disaster Warning and Control in Universities of Yunnan Province, Southwest Forestry University, Kunming, China

**Keywords:** 10 new taxa, Dothideomycetes, phylogeny, taxonomy, saprobic

## Abstract

The Xizang Autonomous Region, China, is a key ecological security barrier, renowned for its rich biodiversity and unique resources. It supports diverse life forms, including fungi, which are vital for ecological balance and the restoration of the region’s ecosystems. This study presents the results of a microfungal survey conducted on the Plateau, focusing on the orders Pleosporales and Kirschsteiniotheliales within the class Dothideomycetes. Based on morphological characteristics and multi-gene (ITS, LSU, SSU, *tef*1, *tub*2, and *rpb*2) phylogeny analyses, we report the discovery of 10 new taxa, including one new genus *Neotriplosphaeria* and nine new species, *Kirschsteiniothelia linzhiensis*, *K*. *yadongensis*, *Neotriplosphaeria yadongensis*, *Periconia linzhiensis*, *Tetraploa linzhiensis*, *Torula dingjieensis*, *T*. *yadongensis*, *Trichobotrys motuoensis*, *Triseptatospora yadongensis*, and one new host record *Periconia spodiopogonis*. The findings provide valuable insights into the morphological and phylogenetic relationships of these fungi, highlighting the Plateau’s significant role in fungal diversity. This study enriches our understanding of fungal biodiversity in extreme environments and underscores the importance of continued exploration in this region.

## 1. Introduction

Xizang Autonomous Region, China, as an important national ecological security barrier, is one of the richest regions in the world in terms of biodiversity and unique biological resources. It is called a rare “green gene bank” and “natural science museum” in the world (Wang et al. 2023b). It provides a good living and reproduction space for all kinds of animals, plants, and fungi (Yang et al. 2017; Zhan et al. 2022). As a large group of eukaryotic organisms, fungi are a crucial part of the biodiversity of the plateau and play an important role in the ecological balance and restoration of the region (Yang et al. 2010). In the past years, especially from 2010 to 2024, researchers have carried out large-scale investigations and research on fungi in the Qinghai-Xizang Plateau, and have achieved gratifying and important results (Huang et al. 2015; Su et al. 2015; Ren et al. 2016; Timdal et al. 2016; Zhao et al. 2016; Chen et al. 2017, 2020; Shi et al. 2018; Pem et al. 2019, 2024; Phookamsak et al. 2019; Hyde et al. 2020, 2021; Boonmee et al. 2021; Chethana et al. 2021; Liu et al. 2022; Ma et al. 2023, 2025; Xu et al. 2023a, 2023b, 2024a, 2024b; He et al. 2024a; Wang et al. 2024a, 2024b; Phurbu et al. 2024). However, the remarkable species profusion within the region implies that a considerable number of fungi have yet to be identified.

Pleosporales, one of the largest orders within the class Dothideomycetes, was formally introduced by Barr (1987). It is a highly diverse order, currently encompassing 91 families and 653 genera (Hyde et al. 2024b). Members of Pleosporales are primarily characterised by their production of perithecial ascomata, which are flask-shaped fruiting bodies that typically possess a papillate, ostioles, the asexual morph being mainly coelomycetous and hyphomycetous (Hyde et al. 2013; Hongsanan et al. 2020; Pem et al. 2024). Pleosporales fungi have a global distribution in various ecosystems, ranging from terrestrial to aquatic environments (Hongsanan et al. 2020). They exhibit a high abundance in temperate regions and have been identified in soils, decaying wood, and plant debris (Hongsanan et al. 2020). In addition, some species of Pleosporales were reported as plant pathogens that can cause diseases in economically important crops, such as *Alternaria*, *Bipolaris*, *Leptosphaeria*, *Phaeosphaeria*, and *Pyrenophora* (Tanaka et al. 2015; El-Demerdash 2018; Jayawardena et al. 2019a, 2019b, 2022, 2025; Bhunjun et al. 2020). Kirschsteiniotheliales was introduced by Hernández-Restrepo et al. (2017) based on phylogenetic analysis to accommodate Kirschsteiniotheliaceae, with *Kirschsteiniothelia* as the type genus. Kirschsteiniotheliales currently encompasses one family and two *incertae sedis* genera (Hyde et al. 2024b).

Supported by the Second Tibetan Plateau Scientific Expedition and Research Program, seven field trips were carried out in the southeast of Xizang Autonomous Region, China (He et al. 2023, 2024b; Thiyagaraja et al. 2024) and more than 26 specimens of Pleosporales and Kirschsteiniotheliales were collected. In this paper, we used morphological data together with phylogenetic analysis of the internal transcribed spacer region (ITS), the large subunit rDNA (LSU), the beta-tubulin (*tub*2), the translation elongation factor 1-α (*tef*1) gene, and RNA polymerase II second-largest subunit (*rpb*2) to assess the systematic position of these specimens.

## 2. Materials and methods

### 2.1. Collection, fungal isolation, and morphology

Specimens were collected from Xizang Autonomous Region, China, following the methods of He et al. (2024b). The specimens were observed under a NIKON MODEL C-PSN Series dissecting stereo microscope (Nikon, Japan). Using a surgical needle transferred into a clean slide and photographed with the NIKON ECLIPSE Ni-U microscope imaging system (Nikon, Japan), observations of conidiophores, conidiogenous cells, and conidia were carried out. A photoplate was made using Adobe Photoshop CC 2019. The fungal structures were measured using Image Frame Work software. Pure cultures were obtained via single spore isolation using potato dextrose agar (PDA) as described in Senanayake et al. (2020) and incubated at 25 °C for one week. The pure cultures were deposited at the Kunming Institute of Botany Culture Collection (KUNCC). Specimens were deposited at the Herbarium of Cryptogams Kunming Institute of Botany, Chinese Academy of Sciences, Kunming, China (KUN-HKAS). Facesoffungi and Index Fungorum numbers were registered following the protocol described in Jayasiri et al. (2015) and Index Fungorum in 2025, respectively.

### 2.2. DNA extraction, PCR amplification, and sequencing

The mycelia growing on a Potato Dextrose Agar (PDA) plate were used to extract DNA using the Trilief™ Plant Genomic DNA Kit (Tsingke Biological Technology Co., Ltd. in Beijing, China). When the fungi did not germinate or grow in culture, DNA was directly extracted using a DNA extraction kit (E.Z.N.A.® Forensic DNA kit, D3591-01, Omega Bio-Tek), following the manufacturer instructions (Telle and Thines 2008). The primer pairs ITS5/ITS4 (White et al. 1990), NS1/NS4 (White et al. 1990), LR0R/LR5 (Vilgalys and Hester 1990), T1/T22 (O’Donnell and Cigelnik 1997), EF1-983F/EF1-2218 R (Carbone and Kohn 1999), and fRPB2-5F/fRPB2-7cR (Liu et al. 1999) were used for amplification of the internal transcribed spacer region ITS1-5.8S-ITS2 (ITS), the large subunit rDNA (LSU), the beta-tubulin (*tub*2), the translation elongation factor 1-α (*tef*1) gene, and RNA polymerase II second-largest subunit (*rpb*2), respectively. The polymerase chain reaction (PCR) reaction was performed in a 25 μL reaction volume, comprising 21 μL PCR Mix (2× Rapid *Taq* Master Mix, Vazyme Biotech Co., Ltd., Nanjing, China), 1 μL of each primer, 2 μL DNA template (Table 1). The PCR products were visualised using agarose gel electrophoresis. The materials together with the targeted bands were sent to Sangon Biotech Co. Ltd., Kunming, China, for sequencing. The newly generated sequences were submitted to the GenBank (Tables 2–7).

**Table 1.** Locus, primers, and PCR amplification conditions used in this study.

Locus	Primers	PCR amplification conditions
SSU	NS1/NS4	95 °C: 5 min, (95 °C: 15 s, 55 °C: 15 s, 72 °C: 15 s) × 40 cycles
ITS	ITS5/ITS4	
LSU	LR0R/LR5	
*tef*1	EF1-983F/EF1-2218R	95 °C: 5 min, (95 °C: 45 s, 52 °C: 45 s, 72 °C: 70 s) × 35 cycles
*rpb*2	fRPB2-5F/fRPB2-7cR	95 °C: 5 min, (95 °C: 45 s, 55 °C: 120 s, 72 °C: 50 s) × 35 cycles

**Table 2.** GenBank accession numbers of the taxa in this study of *Kirschsteiniothelia*.

Taxon	Voucher	GenBank accession numbers		
		ITS	LSU	SSU
***Kirschsteiniothelia acutispora***	**MFLU21-0127**	**OP120780**	**ON980758**	**ON980754**
*Kirschsteiniothelia aethiops*	MFLUCC 16-1104	MH182583	MH182589	MH182615
*Kirschsteiniothelia aethiops*	MFLUCC 15-0424	KU500571	KU500578	KU500585
***Kirschsteiniothelia aquatica***	**MFLUCC 16-1685**	**MH182587**	**MH182594**	**MH182618**
***Kirschsteiniothelia arasbaranica***	**IRAN 2508C**	**KX621983**	**KX621984**	**KX621985**
***Kirschsteiniothelia atra***	**DEN**	**MG602687**	N/A	N/A
***Kirschsteiniothelia bulbosapicalis***	**GZCC 23-0732**	**PQ248937**	**PQ248933**	**PQ248929**
***Kirschsteiniothelia cangshanensis***	**MFLUCC 16-1350**	**MH182584**	**MH182592**	N/A
***Kirschsteiniothelia chiangmaiensis***	**MFLUCC 23-0209**	**OR575473**	**OR575474**	**OR575475**
***Kirschsteiniothelia crustacea***	**MFLU21-0129**	**MW851849**	**MW851854**	N/A
***Kirschsteiniothelia dendryphioides***	**KUNCC 10431**	**OP626354**	**PQ248935**	**PQ248931**
***Kirschsteiniothelia distoseptata***	**HKAS 131382**	**OR714747**	N/A	**OR743200**
***Kirschsteiniothelia dujuanhuensis***	**HKAS 129177**	**OQ874971**	**OQ732682**	**OQ875039**
*Kirschsteiniothelia dushanensis*	GZCC19-0415	OP377845	MW133830	MW134610
*Kirschsteiniothelia ebriosa*	CBSH-23379	N/A	LT985885	N/A
***Kirschsteiniothelia emarceis***	**FLU10-0037**	**NR_138375**	**NG059454**	N/A
***Kirschsteiniothelia extensum***	**MFLU21-0130**	**MW851850**	**MW851855**	N/A
***Kirschsteiniothelia fluminicola***	**MFLUCC 16-1263**	**MH182582**	**MH182588**	N/A
***Kirschsteiniothelia guangdongensis***	**ZHKUCC 22-0233**	**OR164946**	**OR164974**	N/A
***Kirschsteiniothelia guizhouensis***	**GZCC 24-0034**	**PQ404852**	**PQ404856**	**PQ404859**
***Kirschsteiniothelia inthanonensis***	**MFLUCC 23-0277**	**OR762773**	**OR762781**	**OR764784**
***Kirschsteiniothelia laojunensis***	**KUN.L 88727**	**PP081651**	**PP081658**	N/A
***Kirschsteiniothelia lignicola***	**MFLUCC 10-0036**	**HQ441567**	**HQ441568**	**HQ441569**
***Kirschsteiniothelia linzhiensis***	HKAS 144539*	**PV484695**	**PQ675402**	**PQ675362**
*Kirschsteiniothelia linzhiensis*	HKAS 144540*	PV484696	PQ675403	PQ675363
***Kirschsteiniothelia longiconidiophora***	**KUNCC:23-13756**	**OR589303**	**OR600952**	**OR743201**
***Kirschsteiniothelia longirostrata***	**GZCC 23-0733**	**PQ248939**	**PQ248934**	**PQ248930**
***Kirschsteiniothelia longispora***	**HKAS 136268**	**PQ038266**	**PQ038273**	**PQ046108**
***Kirschsteiniothelia nabanheensis***	**HJAUP C2004**	**OQ023197**	**OQ023273**	**OQ023038**
***Kirschsteiniothelia phoenicis***	**MFLUCC 18-0216**	**MG859978**	**MG860484**	**MG859979**
***Kirschsteiniothelia pini***	**UESTCC24.0131**	**PP835321**	**PP835315**	**PP835318**
***Kirschsteiniothelia puerensis***	**ZHKUCC:22-0271**	N/A	**NG_242017**	**OP451020**
***Kirschsteiniothelia ramus***	**GZCC:23-0596**	**OR098711**	**OR091333**	N/A
***Kirschsteiniothelia rostrata***	**MFLUCC 15-0619**	**KY697280**	**KY697276**	**KY697278**
***Kirschsteiniothelia saprophytica***	**MFLUCC_23-0275**	**OR762774**	**OR762783**	N/A
***Kirschsteiniothelia septemseptata***	**FLU21-0126**	**OP120779**	**ON980757**	**ON980752**
***Kirschsteiniothelia sichuanensis***	**HKAS 134908**	**PP785368**	**PP784322**	N/A
*Kirschsteiniothelia* sp.	CX79B2	OQ645272	OQ645286	OQ645279
*Kirschsteiniothelia* sp.	SICAUCC 23-0043	PP060659	PP057952	PP003820
*Kirschsteiniothelia* sp.	SICAUCC 23-0044	PP060660	PP057953	PP003821
*Kirschsteiniothelia* sp.	HJAUP C1209	PP505546	PP506568	PP527763
*Kirschsteiniothelia* sp.	HJAUP C1501	PP505547	PP506569	N/A
*Kirschsteiniothelia* sp.	HJAUP C1273	PP505548	PP506566	PP506565
*Kirschsteiniothelia* sp.	HJAUP C1313	PP505549	PP506562	PP506563
*Kirschsteiniothelia* sp.	KUNCC:23-13755	N/A	N/A	OR743199
***Kirschsteiniothelia spatiosum***	**MFLU21-0128**	**OP077294**	N/A	**ON980753**
***Kirschsteiniothelia submersa***	**MFLUCC 15-0427**	**KU500570**	**KU500577**	**KU500584**
*Kirschsteiniothelia tectonae*	MFLUCC 12-0050	KU144916	KU764707	N/A
***Kirschsteiniothelia thailandica***	**MFLUCC 20-0116**	**MT985633**	**MT984443**	**MT984280**
*Kirschsteiniothelia thujina*	JF13210	KM982716	KM982718	KM982717
***Kirschsteiniothelia vinigena***	**CBSH-23378**	N/A	**NG075229**	N/A
***Kirschsteiniothelia weiningensis***	**GZCC 24-0072**	**PQ404851**	**PQ404855**	**PQ404858**
***Kirschsteiniothelia xishuangbannaensis***	**ZHKUCC 22-0220**	**OP289566**	**OP303181**	**OP289564**
***Kirschsteiniothelia xishuiensis***	**GZCC 24-0052**	**PQ404850**	**PQ404854**	**PQ404857**
***Kirschsteiniothelia yadongensis***	HKAS 144543*	**PQ684985**	**PV483694**	**PV483715**
*Kirschsteiniothelia yadongensis*	HKAS 144544*	PQ684986	PV483695	PV483716
***Kirschsteiniothelia zizyphifolii***	**MFLUCC 23-0270**	**OR762768**	**OR762776**	**OR764779**
*Homortomyces combreti*	CPC 19808	JX517281	JX517291	N/A
***Homortomyces tamaricis***	**MFLUCC 13–0441**	**NR_155161**	**NG_059495**	N/A

The newly generated sequences are shown in gray shade, and the type strains are marked with an asterisk (*). Other type strains are in bold. ‘N/A’ indicates that the data are not available in GenBank.

**Table 3.** GenBank accession numbers of the taxa in this study of Astrosphaeriellaceae.

Taxon	Voucher	GenBank accession numbers		
		LSU	SSU	*tef*1
*Acrocordiopsis patilii*	MFLU 18-0533	MN017850	MN017916	N/A
***Acrocordiopsis patilii***	**BCC 28166**	**GU479772**	**GU479736**	N/A
***Acuminatispora palmarum***	**MFLU:18-1068**	**MH390437**	**MH390401**	**MH399248**
*Acuminatispora palmarum*	MFLU:18-1069	MH390438	MH390402	MH399249
***Aquatospora cylindrica***	**MFLUCC 18-1287**	**MN913715**	**MT864327**	**MT954375**
***Astrosphaeriella bambusae***	**MFLU:12-2471**	**KT955461**	N/A	**KT955424**
***Astrosphaeriella fusispora***	**MFLUCC 10-0555**	**KT955462**	**KT955443**	**KT955425**
***Astrosphaeriella neofusispora***	**MFLU:11-0197**	**KT955463**	**KT955444**	**KT955426**
*Astrosphaeriella stellata*	MFLU 18-2030	JN846723	JN846733	N/A
***Astrosphaeriella thailandica***	**MFLU:11-0227**	**KT955465**	**KT955445**	**KT955427**
***Astrosphaeriella thysanolaenae***	**MFLU:11-0222**	**KT955466**	**KT955446**	**KT955428**
*Astrosphaeriellopsis bakeriana*	CBS 115556	GU301801	N/A	GU349015
***Astrosphaeriellopsis caryotae***	**MFLU:17-1255**	**NG_058605**	**NG_063660**	**MF588974**
***Caryospora aquatica***	**MFLU:11-1083**	**NG_059058**	**MH057850**	N/A
***Caryospora quercus***	**MFLU:18-2151**	**NG_066440**	**MK347869**	N/A
***Caryospora submersa***	**MFLU:18-1539**	**NG_073802**	N/A	N/A
*Mycopepon smithii*	GMB0202	MH049445	MH049453	N/A
*Mycopepon smithii*	LGS20	MH049442	MH049450	N/A
*Mycopepon smithii*	SMH 1609	AF279400	AF279399	N/A
***Pithomyces caryotae***	**MFLUCC 13-0828**	**MF588999**	**NG_063662**	**MF588978**
*Pithomyces flavus*	MTCC 12224	KP814133	KP814134	N/A
***Pithomyces licualae***	**MFLUCC 17-2031**	**NG_059846**	**NG_063661**	N/A
*Pithomyces esuvius*	MTCC 12224	KP814136	KP814137	N/A
***Pteridiospora arengae***	**MFLUCC 15-0289**	**NG_228976**	**ON650678**	**ON734017**
***Pteridiospora bambusae***	**MFLU:18-0071**	**MG831565**	**MG831566**	**MG833012**
***Pteridiospora chiangraiensis***	**MFLUCC 11-0162**	**KT955480**	**KT955459**	**KT955442**
***Pteridiospora javanica***	**MFLUCC 11-0159**	**KJ742940**	**KJ739607**	**KJ739605**
***Quercicola fusiformis***	**MFUCC 18-0479**	**MK348009**	**MK347898**	**MK360085**
***Quercicola guttulospora***	**MFLU:18-2192**	**MK348010**	**MK347899**	**MK360086**
***Triseptatospora calami***	**MFLUCC 15-0305**	N/A	**ON650705**	**ON734018**
***Triseptatospora yadongensis***	HKAS 134939*	**PQ675404**	**PQ675364**	**PQ671461**
*Triseptatospora yadongensis*	HKAS 134940*	PQ675405	PQ675365	PQ671462
***Xenoastrosphaeriella aquatica***	**CGMCC 3.20683**	**MZ420753**	**MZ420754**	**MZ442701**
*Xenoastrosphaeriella aquatica*	DLUCC 0869	MZ420752	MZ420755	MZ442700
*Xenoastrosphaeriella tornata*	MFLUCC 11-0196	KT955467	KT955447	KT955429
*Xenoastrosphaeriella tornata*	KUMCC 18-0194	MT659668	MT659669	MT653597
***Murispora bromicola***	**MFLUCC 15-0031**	**NG_059595**	**KT305996**	**KT305999**
***Murispora kazachstanica***	**CBS 148424**	**NG_088293**	**NG_081421**	**OK019410**

The newly generated sequences are shown in gray shade, and the type strains are marked with an asterisk (*). Other type strains are in bold. ‘N/A’ indicates that the data are not available in GenBank.

**Table 4.** GenBank accession numbers of the taxa in this study of Dictyosporiaceae.

Taxon	Voucher	GenBank accession numbers			
		ITS	LSU	SSU	*tef*1
***Aquadictyospora clematidis***	**MFLU 172080**	**MT310592**	**MT214545**	**MT226664**	**MT394727**
***Aquadictyospora lignicola***	**MFLUCC 17-1318**	**MF948621**	**MF948629**	N/A	N/A
***Dendryphiella paravinosa***	**CPC26176**	**KX228257**	**KX228309**	N/A	N/A
*Dendryphiella vinosa*	MFLU200444	MT907477	MT907480	N/A	N/A
***Dictyocheirospora aquatica***	**KUMCC 15-0305**	**KY320508**	**KY320513**	**AB787223**	**AB808489**
***Dictyocheirospora bannica***	**KH332**	**LC014543**	**AB807513**	**MH381759**	N/A
*Dictyocheirospora pseudomusae*	yone 234	LC014550	AB807520	AB797230	AB808496
***Dictyosporium alatum***	**ATCC 34953**	**NR_077171**	**DQ018101**	**DQ018080**	N/A
*Dictyosporium elegans*	NBRC 32502	DQ018087	DQ018100	DQ018079	N/A
***Dictyosporium meiosporum***	**MFLUCC 10-0131**	**KP710944**	**KP710945**	**KP710946**	N/A
*Dictyosporium nigroapice*	MFLUCC 17-2053	MH381768	MH381777	MH381762	MH388821
***Dictyosporium olivaceosporum***	**KH375**	**LC014542**	**AB807514**	**AB797224**	**AB808490**
*Dictyosporium pandanicola*	MFLUCC 18-0331	MZ490792	MZ490776	N/A	MZ501208
*Dictyosporium tetrasporum*	KT2865	LC014551	AB807519	AB797229	AB808495
***Dictyosporium tratense***	**MFLUCC 17-2052**	**MH381767**	**MH381776**	**MH381761**	**MF388820**
***Gregarithecium curvisporum***	**KT922**	**AB809644**	**AB807547**	**AB797257**	**AB808523**
*Jalapriya pulchra*	MFLU17-1683	MF948628	MF948636	N/A	MF953171
*Jalapriya toruloides*	CBS209.65	DQ018093	DQ018104	DQ018081	N/A
***Neodendryphiella brassaiopsidis***	**MHZU:23-0113**	**OR365455**	**OR365485**	**OR365491**	N/A
***Neodendryphiella michoacanensis***	**CBS:144323**	**NR_160583**	**NG_066395**	N/A	N/A
***Neodendryphiella tarraconensis***	**CBS:144324**	**NR_160582**	**NG_066394**	N/A	N/A
***Paradictyocheirospora tectonae***	**NFCCI:4878**	**NR_189839**	**NG_241935**	N/A	**MW854832**
***Pseudocoleophoma calamagrostidis***	**KT3284**	**LC014592**	**LC014609**	**LC014604**	**LC014614**
*Pseudocoleophoma flavescens*	CBS178.93	N/A	GU238075	GU238216	N/A
***Pseudocoleophoma polygonicola***	**KT731**	**AB809634**	**AB807546**	**AB797256**	**AB808522**
***Pseudodictyosporium elegans***	**CBS 688.93**	**MH862454**	**MH874101**	**DQ018084**	N/A
***Pseudodictyosporium thailandica***	**MFLUCC 16-0029**	**KX259520**	**KX259522**	**KX259524**	**KX259526**
*Trichobotrys effusa*	JAUCC 6359	PP406377	PP407503	PP407508	PP405621
*Trichobotrys effusus*	SNC73	PP592489	PP621120	PP639248	PP761036
***Trichobotrys meilingensis***	**JAUCC 4985**	**PP406380**	**PP407504**	**PP407509**	**PP405623**
***Trichobotrys motuoensis***	HKAS 144531*	**PQ684987**	**PQ675406**	**PQ675366**	**PQ671463**
*Trichobotrys motuoensis*	HKAS 144532*	PQ684988	PQ675407	PQ675367	PQ671464
***Trichobotrys sinensis***	**KUNCC 23-14554**	**OR233595**	**OR335805**	**OR501421**	**OR547995**
***Trichobotrys yunjushanensis***	**JAUCC 4987**	**PP406378**	**PP407506**	**PP407511**	**PP405622**
***Verrucoccum coppinsii***	**E. 00814291**	**NR_177147**	**NG_153929**	**NG_081399**	N/A
***Verrucoccum spribillei***	**MSC 31786**	**NR_177539**	**NG_088165**	**NG_087876**	N/A
***Vikalpa grandispora***	**KUNCC 22-12425**	**OP526638**	**OP526648**	**OP526628**	**OP542240**
***Vikalpa sphaerica***	**CGMCC 3.20682**	**OP526639**	**OP526649**	**OP526629**	**OP542241**
*Periconia igniaria*	CBS 379.86	LC014585	AB807566	AB797276	AB808542
*Periconia igniaria*	CBS 845.96	LC014586	AB807567	AB797277	AB808543

The newly generated sequences are shown in gray shade, and the type strains are marked with an asterisk (*). Other type strains are in bold. ‘N/A’ indicates that the data are not available in GenBank.

**Table 5.** GenBank accession numbers of the taxa in this study of *Periconia.*

Taxon	Voucher	GenBank accession numbers			
		SSU	ITS	LSU	*tef*1
*Periconia algeriana*	CBS 321.79	N/A	MH861212	MH872979	N/A
*Periconia alishanica*	KUMCC 19-0174	N/A	MW063167	MW063231	MW183792
***Periconia alishanica***	**NCYUCC 19-0186**	N/A	**MW063166**	**MW063230**	**MW183791**
***Periconia ananasi***	**MFLUCC:21-0155**	**OL606142**	**OL753685**	**OL606153**	**OL912946**
***Periconia aquatica***	**MFLUCC16-0912**	N/A	**KY794701**	**KY794705**	**KY814760**
***Periconia arecacearum***	**MFLU 19-0803**	**PP639222**	**PP592462**	**PP621090**	**PP828795**
***Periconia artemisiae***	**KUMCC 20-0265**	**MW448658**	**MW448657**	**MW448571**	**MW460898**
*Periconia atropurpurea*	CBS 381.55	N/A	MH857524	MH869061	N/A
***Periconia banksiae***	**CBS 129526**	N/A	**JF951147**	**NG_064279**	N/A
*Periconia byssoides*	MFLUCC17-2292	MK347858	MK347751	MK347968	MK360069
*Periconia byssoides*	MFLUCC18-1553	MK347914	MK347806	MK348025	MK360068
***Periconia caespitosa***	**LAMIC 110/16**	N/A	**MH051906**	**MH051907**	N/A
***Periconia calamagrostidicola***	**CBS H-25342**	N/A	**NR_197928**	**PP791460**	**PP780620**
***Periconia celtidis***	**MFLU 19-2784**	N/A	**NR_174830**	**NG_079543**	N/A
*Periconia chengduensis*	UESTCC 22.0140	OP956046	OP955977	OP956002	OP961443
***Periconia chiangraiensis***	**MFLU 21-0280**	**OL606143**	**OL753686**	**OL606154**	**OL912947**
*Periconia chimonanthi*	KUMCC 20-0266	MW448656	NR_176752	MW448572	MW460897
*Periconia circinata*	CBS 263.37	N/A	MW810265	MH867413	MW735660
***Periconia citlaltepetlensis***	**ENCB 140251**	N/A	**MH890645**	**MT625978**	N/A
*Periconia citlaltepetlensis*	IOM 325319.2	N/A	MT649221	MT649216	N/A
*Periconia cookei*	UESTCC 22.0134	OP956037	OP955968	OP955993	N/A
*Periconia cortaderiae*	MFLUCC 15-0457	KX986345	KX965732	KX954401	KY310703
***Periconia cynodontis***	**CGMCC 3.23927**	**OP909920**	**OP909925**	**OP909921**	**OP961434**
***Periconia cyperacearum***	**CPC 32138**	N/A	**MH327815**	**MH327851**	N/A
***Periconia delonicis***	**MFLUCC 17-2584**	**MK347832**	N/A	**MK347941**	**MK360071**
***Periconia didymosporum***	**MFLU 15-0058**	**KP761738**	**KP761734**	**KP761731**	**KP761728**
***Periconia digitata***	**CBS 510.77**	**AB797271**	**LC014584**	**AB807561**	**AB808537**
***Periconia elaeidis***	**MFLUCC 17-0087**	**MH108551**	**MG742713**	**MH108552**	N/A
***Periconia endophytica***	**ZHKUCC 23-0995**	**PP277722**	**OR995582**	**OR995588**	**PP025968**
*Periconia epilithographicola*	MFLUCC21-0153	OL606144	OL753687	OL606155	OL912948
***Periconia festucae***	**CGMCC 3.23929**	**OP956042**	**OP955973**	**OP955998**	**OP961439**
***Periconia floridana***	**CBS 150884**		**NR_197917**	**NG_244042**	
*Periconia genistae*	CBS 322.79	N/A	MH861213	MH872980	N/A
***Periconia homothallica***	**CBS 139698**	**AB797275**	**AB809645**	**NG_059397**	**AB808541**
*Periconia igniaria*	CBS 298.66	N/A	MH858798	MH870438	N/A
*Periconia igniaria*	CBS 583.66	N/A	MH858888	MH870553	N/A
*Periconia imperatae*	UESTCC 22.0145	OP956048	OP955979	OP956004	OP961445
***Periconia kunmingensis***	**HKAS:102239**	**OR225814**	**MH892346**	**MH892400**	**MH908963**
*Periconia lateralis*	CBS 292.36	N/A	MH855804	MH867311	N/A
***Periconia linzhiensis***	HKAS 144526*	**PQ675368**	**PQ684989**	**PQ675408**	**PQ671465**
*Periconia linzhiensis*	HKAS 144525*	PQ675369	PQ684990	PQ675409	PQ671466
*Periconia macrospinosa*	CPC 22898	KP184080	KP183999	KP184038	N/A
*Periconia minutissima*	MFLUCC 15-0245	N/A	KY794703	KY794707	N/A
***Periconia muchuanensis***	**HKAS 135150**	**PQ066546**	**PQ067783**	**PQ067698**	**PQ278553**
***Periconia neobrittanica***	**CBS 146062**	N/A	**MN562149**	**MN567656**	N/A
***Periconia neominutissima***	**CBS 149514**	N/A	**NR_189522**	**NG_242103**	N/A
***Periconia palmicola***	**MFLUCC 14-0400**	**MN648319**	N/A	**MN648327**	**MN821070**
***Periconia penniseti***	**CGMCC 3.23928**	**OP956040**	**OP955971**	**OP955996**	**OP961437**
***Periconia philadelphiana***	**CPC 42854**	N/A	**OQ628486**	**OQ629068**	N/A
***Periconia prolifica***	**CBS 209.64**	N/A	**NR_160097**	**MH870050**	N/A
*Periconia pseudobyssoides*	UESTCC 22.0135	OP956034	OP955965	OP955990	OP961431
*Periconia pseudobyssoides*	UESTCC 22.0147	OP956044	OP955975	OP956000	OP961441
*Periconia pseudodigitata*	CBS 139699	AB797274	LC014591	AB807564	AB808540
*Periconia sahariana*	CBS 320.79	N/A	MH861211	MH872978	N/A
***Periconia salina***	**MFLU 19-1235**	**MN017912**	**MN047086**	**MN017846**	N/A
***Periconia shannanensis***	**HKAS 134945**	**PP968558**	**PP968552**	**PP968555**	**PP226770**
***Periconia sichuanensis***	**CGMCC 3.25598**	**PQ066545**	**PQ067867**	**PQ067697**	**PQ278551**
***Periconia spodiopogonis***	**CGMCC 3.23932**	**OP956032**	**OP955963**	**OP955988**	**OP961429**
*Periconia spodiopogonis*	HKAS 135635*	PQ675370	PP952765	N/A	N/A
***Periconia submersa***	**MFLUCC 16-1098**	N/A	**KY794702**	**KY794706**	**KY814761**
***Periconia thailandica***	**MFLUCC 17-0065**	**KY753889**	**KY753887**	**KY753888**	N/A
***Periconia thysanolaenae***	**KUMCC 20-0262**	**NG_081407**	**MW442967**	**MW444850**	**MW460896**
***Periconia variicolor***	**CBS 120374**	N/A	**DQ336713**	N/A	N/A
*Periconia verrucosa*	UESTCC 22.0136	OP956035	OP955966	OP955991	OP961432
***Periconia wurfbainiae***	**ZHKUCC 23-0999**	**PP277726**	**OR995586**	**OR995592**	**PP025972**
***Periconia yangjiangensis***	**ZHKUCC 23-0997**	**PP277724**	**OR995584**	**OR995590**	**PP025970**
*Massarina cisti*	CBS 266.62	FJ795490	LC014568	AB807539	AB808514
***Massarina pandanicola***	**MFLU:18-0004**	**NG_065725**	**MG646958**	**NG_064488**	**MG646986**

The newly generated sequences are shown in gray shade, and the type strains are marked with an asterisk (*). Other type strains are in bold. ‘N/A’ indicates that the data are not available in GenBank.

**Table 6.** GenBank accession numbers of the taxa in this study of Tetraplosphaeriaceae.

Taxon	Voucher	GenBank accession numbers			
		SSU		ITS	LSU
***Aquatisphaeria thailandica***	**MFLUCC 21-0025**	**MW890967**	**MW890969**		**MW890763**
*Aquatisphaeria thailandica*	DLUCC B151	MW890968	N/A		MW890764
*Byssolophis sphaerioides*	IFRDCC 2053	GU296140	N/A		GU301805
***Ernakulamia cochinensis***	**MFLUCC 18-1237**	**MT864326**	**MT627670**		**MN913716**
*Ernakulamia krabiensis*	MFLUCC 18-0237	MK347880	MK347773		MK347990
***Ernakulamia tanakae***	**NFCCI 4615**	N/A	**MN937229**		**MN937211**
***Neotriplosphaeria yadongensis***	HKAS 144527*	**PQ675371**	**PQ684991**		**PQ675410**
*Neotriplosphaeria yadongensis*	HKAS 144528*	PQ675372	PQ684992		PQ675411
***Parapolyplosphaeria thailandica***	**MFLU 15-3273**	N/A	**KU248766**		**KU248767**
***Polyplosphaeria fusca***	**KT1616**	**AB524463**	**AB524789**		**AB524604**
***Polyplosphaeria guizhouensis***	**GZCC 23-0598**		**OR427327**		**OR438888**
***Polyplosphaeria hainanensis***	**GZCC 23-0599**	**OR438285**	**OR427323**		**OR438889**
***Polyplosphaeria nabanheensis***	**KUMCC 16-0151**	**MH260352**	**MH275078**		**MH260312**
***Polyplosphaeria pandanicola***	**KUMCC 17-0180**	**MH260353**	**MH275079**		**MH260313**
***Pseudopolyplosphaeria guizhouensis***	**GZCC 19-0247**	**OR134445**	N/A		N/A
***Pseudotetraploa bambusicola***	**CGMCC 3.20939**	**ON332923**	**ON332915**		**ON332933**
***Pseudotetraploa curviappendiculata***	**HC:4930**	**AB524467**	**AB524792**		**AB524608**
***Pseudotetraploa longissima***	**HC:4933**	**AB524471**	**AB524796**		**AB524612**
***Pseudotetraploa rajmachiensis***	**NFCCI 4618**	N/A	**MN937222**		**MN937204**
***Pseudotetraploa yunnanensis***	**KUNCC 10464**	N/A	**OR449073**		**OR438891**
***Quadricrura bicornis***	**CBS 125427**	**AB524472**	**AB524797**		**AB524613**
***Quadricrura meridionalis***	**CBS 125684**	**AB524473**	**AB524798**		**AB524614**
***Shrungabeeja aquatica***	**MFLUCC 18-0664**	N/A	**MT627722**		**MT627663**
***Shrungabeeja fluviatilis***	**GZCC 20-0505**	**OP377989**	**OP377804**		**OP377903**
*Shrungabeeja longiappendiculata*	BCC 76463	KT376471	KT376474		KT376472
*Shrungabeeja vadirajensis*	MFLUCC 17-2362	N/A	MT627681		MN913685
***Tetraploa aquatica***	**MFLU 19-0995**	N/A	**MT530448**		**MT530452**
*Tetraploa aristata*	CBS 996.70	AB524486	AB524805		AB524627
***Tetraploa bambusae***	**KUMCC 21-0844**	**ON077073**	**ON077078**		**ON077067**
*Tetraploa cylindrica*	KUMCC 20-0205	MT893203	MT893205		MT893204
***Tetraploa cylindrica***	**ZHKUCC 22-0087**	**ON555690**	**ON555689**		**ON555688**
***Tetraploa dashaoensis***	**KUMCC 21-0010**	**OL473556**	**OL473549**		**OL473555**
***Tetraploa dwibahubeeja***	**NFCCI 4621**	N/A	**MN937226**		**MN937208**
***Tetraploa endophytica***	**CBS 147114**	N/A	**KT270279**		**MW659165**
***Tetraploa hainanensis***	**GZCC 23-0601**	**OR438286**	**OR427325**		**OR438892**
*Tetraploa juncicola*	CBS 149046	N/A	ON603780		ON603800
*Tetraploa lignicola*	KUNCC 10794	ON422300	ON422286		ON422294
***Tetraploa*** ***linzhiensis***	HKAS 144535*	**PQ675373**	PQ684993		**PQ675412**
*Tetraploa linzhiensis*	HKAS 144536*	PQ675374	PQ684994		PQ675413
***Tetraploa nagasakiensis***	**KT 1682**	**AB524489**	**AB524806**		**AB524630**
***Tetraploa obpyriformis***	**KUMCC 21-0011**	**OL473557**	**OL473558**		**OL473554**
***Tetraploa oryzae***	**MFLU 23-0195**	**OR458352**	**OR438372**		**OR438841**
***Tetraploa palmae***	**HKAS 115638**	**PP639245**	N/A		**PP621116**
***Tetraploa phoenicis***	**HKAS 115675**	**PP639246**	**PP592486**		**PP621117**
***Tetraploa pseudoaristata***	**NFCCI 4624**	N/A	**MN937232**		**MN937214**
***Tetraploa puzheheiensis***	**KUMCC 20-0151**	N/A	**MT627744**		**MT627655**
***Tetraploa sasicola***	**KT 563**	**AB524490**	**AB524807**		**AB524631**
*Tetraploa scheueri*	CY112	N/A	HQ607964		N/A
*Tetraploa* sp.	HKAS 131566	N/A	PP768923		PP768935
***Tetraploa thailandica***	**MFLUCC 21-0030**	**MZ413274**	**MZ412518**		**MZ412530**
***Tetraploa thrayabahubeeja***	**NFCCI 4627**	N/A	**MN937235**		**MN937217**
*Tetraploa wurfbainiae*	MHZU 23-0231	OR626043	OR626039		OR626041
***Tetraploa yakushimensis***	**KT 1906**	**AB524491**	**AB524808**		**AB524632**
***Tetraploa yunnanensis***	**MFLUCC 19-0319**	**MT864341**	**MT627743**		**MN913735**
***Triplosphaeria acuta***	**KT 1170**	**AB524492**	**AB524809**		**AB524633**
***Triplosphaeria cylindrica***	**KT 1800**	**AB524494**	**AB524810**		**AB524635**
***Triplosphaeria maxima***	**KT 870**	**AB524496**	**AB524812**		**AB524637**
***Murispora aquatica***	**MFLU 19-0990**	**MN325077**	**MN325085**		**MN325075**
***Murispora cardui***	**MFLU 15-2248**	**NG_063589**	**NR_171721**		**KT709176**

The newly generated sequences are shown in gray shade, and the type strains are marked with an asterisk (*). Other type strains are in bold. ‘N/A’ indicates that the data are not available in GenBank.

**Table 7.** GenBank accession numbers of the taxa in this study of *Torula.*

Taxon	Voucher	GenBank accession numbers				
		ITS	LSU	SSU	*rpb*2	*tef*1
***Torula acaciae***	**CPC 29737**	**KY173471**	**KY173560**	N/A	**KY173594**	N/A
***Torula aquatica***	**MFLUCC 16-1115**	**MG208167**	**MG208146**	N/A	**MG207977**	N/A
*Torula aquatica*	DLUCC 0550	MG208166	MG208145	N/A	MG207976	MG207996
***Torula breviconidiophora***	**KUMCC 18-0130**	**MK071670**	**MK071672**	**MK071697**	N/A	**MK077673**
***Torula calceiformis***	**HKAS 125551**	**OP751055**	**OP751053**	**OP751051**	**OQ630510**	**OQ630512**
*Torula calceiformis*	HKAS 125552	OP751054	OP751052	OP751050	OQ630511	OQ630513
***Torula camporesii***	**KUMCC 19-0112**	**MN507400**	**MN507402**	**MN507401**	**MN507404**	**MN507403**
***Torula chiangmaiensis***	**KUMCC 16-0039**	**MN061342**	**KY197856**	**KY197863**	N/A	**KY197876**
***Torula chinensis***	**UESTCC 22-0085**	**OQ127986**	**OQ128004**	**OQ127995**	N/A	N/A
***Torula chromolaenae***	**KUMCC 16-0036**	**MN061345**	**KY197860**	**KY197867**	**KY197873**	**KY197880**
*Torula chromolaenae*	MFLUCC 20-0237	MW412524	MW412518	MW412515	MW422161	MW422158
***Torula dingjieensis***	HKAS 144529*	**PQ684995**	**PQ675414**	**PQ675375**	**PQ671471**	**PQ671467**
*Torula dingjieensis*	HKAS 144530*	PQ684996	PQ675415	PQ675376	PQ671472	PQ671468
***Torula fici***	**CBS 595.96**	**KF443408**	**KF443385**	**KF443387**	**KF443395**	**KF443402**
*Torula fici*	KUMCC 16-0038	MN061341	KY197859	KY197866	KY197872	KY197879
***Torula gaodangensis***	**MFLUCC 17-0234**	**MF034135**	**NG_059827**	**MF034134**	N/A	N/A
***Torula goaensis***	**MTCC 12620**	**NR_159045**	**NG_060016**	N/A	N/A	N/A
***Torula herbarum***	**CPC 24414**	**KR873260**	**KR873288**	N/A	N/A	N/A
***Torula hollandica***	**CBS 220.69**	**NR_132893**	**NG_064274**	**KF443389**	**KF443393**	**KF443401**
***Torula hydei***	**KUMCC 16-0037**	**MN061346**	**MH253926**	**MH253928**	N/A	N/A
***Torula lancangjiangensis***	**HKAS 112709**	**NR_175706**	**NG_081516**	**NG_078759**	**MW729780**	**MW729785**
*Torula lancangjiangensis*	B03	MZ538529	MZ538563	N/A	N/A	MZ567104
***Torula longan***	**ZHKUCC 22-0121**	**OR194035**	**OR194027**	**OR194032**	**OR228535**	**OR228537**
*Torula longan*	ZHKUCC 22-0122	OR194036	OR194028	OR194033	OR228536	OR228538
***Torula luguhuensis***	**KUNCC 22-12427**	**OQ729758**	**OQ947766**	N/A	**OQ999002**	**OQ999004**
***Torula mackenziei***	**MFLUCC 13-0839**	**MN061344**	**KY197861**	**KY197868**	**KY197874**	**KY197881**
*Torula mackenziei*	HKAS 112705	MW723058	MW879525	MW774581	N/A	N/A
***Torula masonii***	**CBS 245.57**	**NR_145193**	**NG_058185**	N/A	N/A	N/A
*Torula masonii*	MFLUCC 20-0239	MW412523	MW412517	MW412514	MW422160	MW422157
***Torula phytolaccae***	**ZHKUCC 22-0107**	**ON611796**	**ON611800**	**ON611798**	**ON660879**	**ON660881**
*Torula phytolaccae*	ZHKUCC 22-0108	ON611795	ON611799	ON611797	ON660878	ON660880
***Torula pluriseptata***	**MFLUCC 14-0437**	**MN061338**	**KY197855**	**KY197862**	**KY197869**	**KY197875**
*Torula polyseptata*	MFLUCC 17-1495	MT214382	MT214476	MT214427	MT235830	MT235791
***Torula sichuanensis***	**UESTCC 22-0087**	**OQ127981**	**OQ127999**	**OQ127990**	N/A	N/A
***Torula snbmersa***	**UESTCC 22-0086**	**OQ127985**	**OQ128003**	**OQ127994**	N/A	N/A
***Torula suae***	**CGMCC 3.24259**	**OP359406**	**OP359415**	**OP369300**	**OP476730**	**OP471618**
*Torula sundara*	UESTCC 22-0125	OQ127984	OQ128002	OQ127993	N/A	N/A
*Torula sundara*	UESTCC 22-0088	OQ127983	OQ128001	OQ127992	N/A	N/A
*Torula sundara*	MFLU 21-0089	OM276824	OM287866	N/A	N/A	N/A
***Torula thailandica***	**MFLU:19-2856**	**MN907426**	**MN907427**	**MN907428**	N/A	N/A
***Torula yadongensis***	HKAS 144533*	**PQ684997**	**PQ675416**	**PQ675377**	**PQ671473**	**PQ671469**
*Torula yadongensis*	HKAS 144534*	PQ684998	PQ675417	PQ675378	PQ671474	PQ671470
***Cylindrotorula indica***	**NFCCI:4836**	**MT339444**	**MT339442**	N/A	**MT321490**	**MT321492**
*Cylindrotorula indica*	NFCCI:4837	MT339445	MT339443	N/A	MT321491	MT321493

The newly generated sequences are shown in gray shade, and the type strains are marked with an asterisk (*). Other type strains are in bold. ‘N/A’ indicates that the data are not available in GenBank.

### 2.3. Phylogenetic analyses

The raw sequences were assembled using SeqMan (Katoh et al. 2005). Comparison with GenBank data was conducted to identify their close relatives. The assembled sequences were compared with GenBank data to identify their closest related taxa. Each gene matrix was aligned separately using MAFFT v. 6.8 (Katoh et al. 2005). The aligned datasets were then manually edited with BioEdit v. 7.0.9 (Hall 1999). SequenceMatrix v. 1.7.8 (Vaidya et al. 2011) was used to combine these datasets. The combined alignment was subsequently used for maximum likelihood (ML) and Bayesian inference (BI) analyses.

The ML analysis was performed using RAxML-HPC2 on XSEDE v. 8.2.10 on the CIPRES Science Gateway v. 3.3 (Miller et al. 2010) with default parameters. The adjustments were made by setting the bootstrap iterations to 1,000, and the substitution model was defined as GTR+GAMMA+I. MrModeltest v. 2.3 was utilised to determine the optimal model for each gene. The BI analysis was conducted using MrBayes v. 3.2.6 (Ronquist et al. 2012). Six simultaneous Markov Chain Monte Carlo (MCMC) chains ran for 500,000 generations, and trees were sampled every 1,000th generation until the standard deviation of the split frequencies dropped below 0.01. The first 25% of generations were discarded as the burn-in phase (Huelsenbeck and Ronquist 2001). The remaining ones were used to calculate Posterior probabilities (PP). The resulting phylogenetic tree was visualised using FigTree v. 1.4.0 and further edited using Adobe Illustrator 2020 and Adobe Photoshop CC 2019.

## 3. Results

**Kirschsteiniotheliales** Hern-Restr., R.F. Castañeda, Gené & Crous

**Kirschsteiniotheliaceae** Boonmee & K.D. Hyde

**Figure 1.**
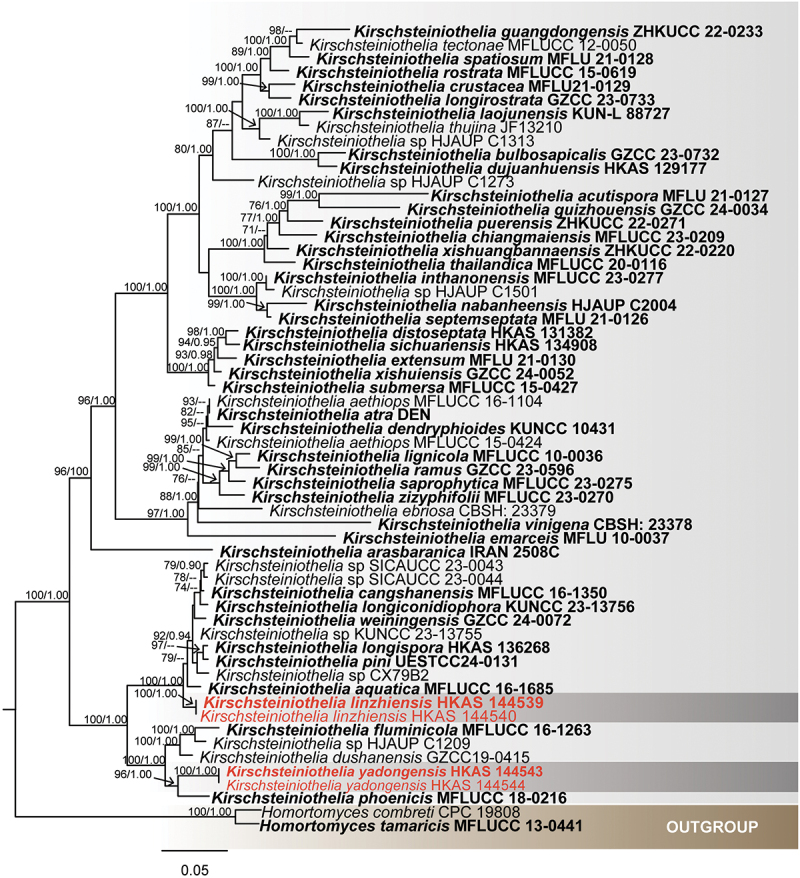
Maximum likelihood tree of *Kirschsteiniothelia* from a four-locus dataset (ITS, SSU, and LSU). RAxML bootstrap support values (ML ≥ 70%) and Bayesian posterior probability (PP ≥ 0.90) are shown at the nodes (ML/PP). The scale bar indicates 0.05 changes per site. *Homortomyces combreti* (CPC 19808) and *H. tamaricis* (MFLUCC 13-0441) were selected as outgroup taxa. The type strains are in bold while the newly generated sequences are indicated in red.

For *Kirschsteiniothelia*, 56th taxa were used (Table 2) to assess the taxonomic placement of new isolates using combined three loci (SSU: 1–926 bp; LSU: 927–1,714 bp; ITS: 1,715–2,168 bp), with *Homortomyces combreti* and *H. tamaricis* (Wijayawardene et al. 2014) as the outgroup. The ML and BI methods were used to obtain the phylogenetic results (Figure 1). The total character number of combined sequences was 2,168 including gaps. The matrix had 909 distinct alignment patterns, with 22.74% of undetermined characters or gaps. Estimated base frequencies were as follows: A = 0.229197, C = 0.252822, G = 0.299630, T = 0.218352; substitution rates: AC = 1.435134, AG = 2.861902, AT = 0.965679, CG = 1.486506, CT = 6.142113, GT = 1.000000; gamma distribution shape parameter α = 0.532956. The best-scoring RAxML tree had a final likelihood value of −15,870.140997. The ML tree topology was similar to the one inferred from BI analysis. Our specimens *Kirschsteiniothelia linzhiensis* (HKAS 144539 & HKAS 144540) and *K*. *yadongensis* (HKAS 144543 & HKAS 144544) formed distinct monophyletic clades with *K. aqutica* and *K. phoenicis* with support value of (100% ML/1.00 PP), respectively.

***Kirschsteiniothelia*** D. Hawksw., Bot. J. Linn. Soc. 91: 182 (1985)

*Kirschsteiniothelia* was introduced by Hawksworth (1985) and was initially classified under Pleosporaceae with *Kirschsteiniothelia aethiops* as the type species, which was later moved to Pleomassariaceae by Barr (1987) based on morphology. Molecular analyses by Kodsueb et al. (2006) further indicated that *K. aethiops* did not belong to Pleosporaceae. Later, Boonmee et al. (2012) proposed a new family, Kirschsteiniotheliaceae (Pleosporales), based on morphological and phylogenetic analyses (ITS, SSU, and LSU) to accommodate *Kirschsteiniothelia* (Boonmee et al. 2012). Subsequently, *Kirschsteiniothelia* was placed in the newly proposed order Kirschsteiniotheliales (Dothideomycetes) by Hernández-Restrepo et al. (2017). Morphologically, the sexual morph of *Kirschsteiniothelia* is characterised by brown, semi-immersed, subglobose ascomata, cylindrical-clavate, bitunicate, 8-spored asci, brown ascospores with smooth walls and usually have 1–2-septate (Hawksworth 1985; Yang et al. 2010; Xu et al. 2023a; Louangphan et al. 2024), and the asexual morph is characterised by macronematous, branched or unbranched, erect, septate, conidiophores, terminal, determinate, monophialidic, conidiogenous cells and cylindric-obclavate, septate, brown to dark brown conidia (Boonmee et al. 2012; Bao et al. 2018). The genus *Kirschsteiniothelia* currently comprises 43 species (Senanayake et al. 2023; Xu et al. 2023a; Louangphan et al. 2024; Tang et al. 2024). The members of *Kirschsteiniothelia* are commonly saprobic on decaying wood (Senanayake et al. 2023). *Kirschsteiniothelia* species are globally distributed and found in a wide range of habitats across both temperate and tropical regions (Suetrong et al. 2009; Boonmee et al. 2012; Jayawardena et al. 2022). In this study, we introduce two new species from the Xizang Autonomous Region, China, based on phylogenetic and morphological analyses.

**Figure 2.**
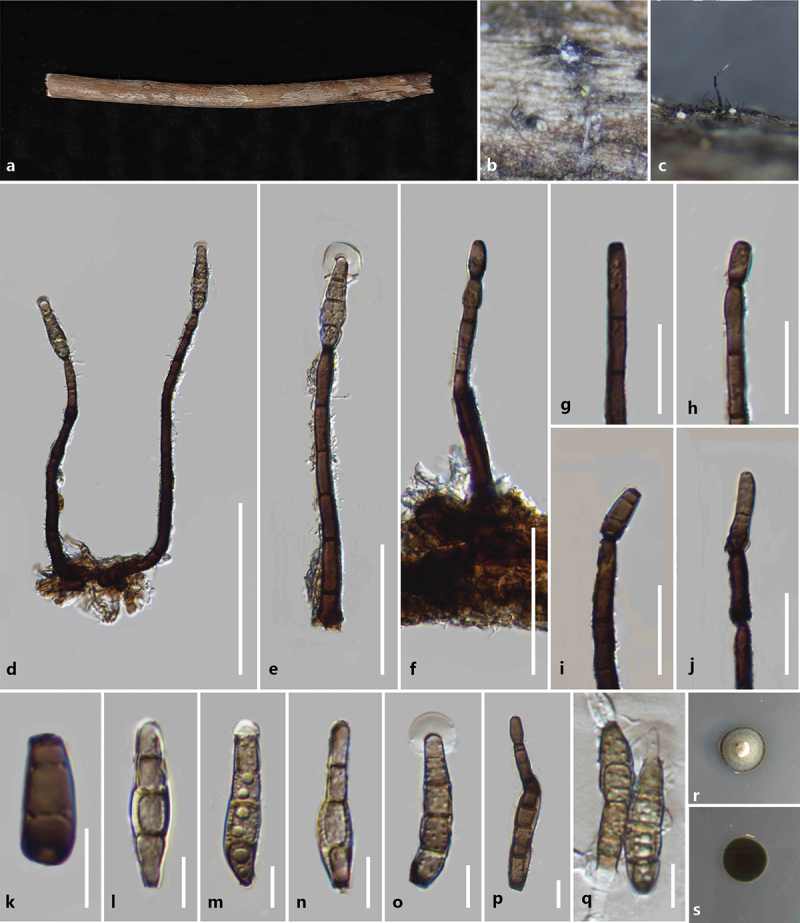
*Kirschsteiniothelia linzhiensis* (HKAS 144539, holotype). (a) Host substrate. (b – c) Colonies on natural substrate. (d – f) Conidiophores. (g – j) Conidiogenous cells and conidia. (k – p) Conidia. (q) Germinated conidium. (r, s) Culture on PDA. Scale bars: d = 100 μm, e – f = 50 μm, g – j = 25 μm, k – q = 10 μm.

***Kirschsteiniothelia linzhiensis*** S.C. He, Q. Zhao & K.D. Hyde, sp. nov. Figure 2

*MycoBank number*: MB857898; *Facesoffungi number*: FoF17072.

*Etymology* – Referring to the collecting site of the type specimen, Linzhi City, Xizang Autonomous Region, China.

*Description* – *Saprobic* on unidentified decaying wood. **Asexual morph**: Hyphomycetous. *Colonies* effuse, scattered, erect, and brown. *Conidiophores* 77–200 × 6–8 μm ($\overset{ˉ}{x}$ = 140 × 7 μm, *n* = 20), macronematous, mononematous, erect, simple, straight or flexuous, unbranched, cylindrical, smooth, thick-walled, septate, pale brown to brown. *Conidiogenous cells* monoblastic, terminal, cylindrical, brown conidia developing at the apex. *Conidia* 31–43 × 6–10 μm ($\overset{ˉ}{x}$ = 36 × 8 μm, *n* = 30), solitary, acrogenous dry, obovoid, guttulate, thick-walled, 3–4-septate, straight or curved, pale brown to brown, with a gelatinous sheath at the apex. **Sexual morph**: Not observed.

*Culture characteristics* – germinating within 24 h on PDA at 25 °C, slow growing, reaching 1.1–1.3 cm after 10 days incubation, above colony grey, reverse black, entire, convex, surface smooth, dense, no pigment.

*Host* – On unknown substrate.

*Known distribution* – China, Xizang Autonomous Region, Linzhi City, Chayu County.

*Material examined* – China, Xizang Autonomous Region, Linzhi City, Chayu County (28°54′N, 96°98′E, 1,878 m), on unknown wood, 13 August 2023, Shucheng He, WZY20 (KUN-HKAS 144539, **holotype**) – ex-type, KUNCC24-18549.

*Notes* – *Kirschsteiniothelia linzhiensis* formed a monophyletic clade *K. aquatica* with 100% ML and 1.00 PP support. Morphologically, *K. linzhiensis* resembles *K. aquatica* in having macronematous, simple, straight or flexuous, unbranched, thick-walled, smooth, septate conidiophores, and solitary, septate conidia (Bao et al. 2018). However, *K. linzhiensis* differs from *K. aquatica* in having obovoid, guttulate, and pale brown to brown conidia with a gelatinous sheath (Bao et al. 2018). The nucleotide differences for ITS, LSU, and SSU between *K. linzhiensis* and *K. aquatica* are 36/762 (4.7%, without gaps), 6/750 (0.8%, without gaps), and 5/740 (0.7%, without gaps), respectively. Based on morphological and phylogenetic analyses, we propose a new species *K. linzhiensis* from Linzhi City, Xizang Autonomous Region, China.

**Figure 3.**
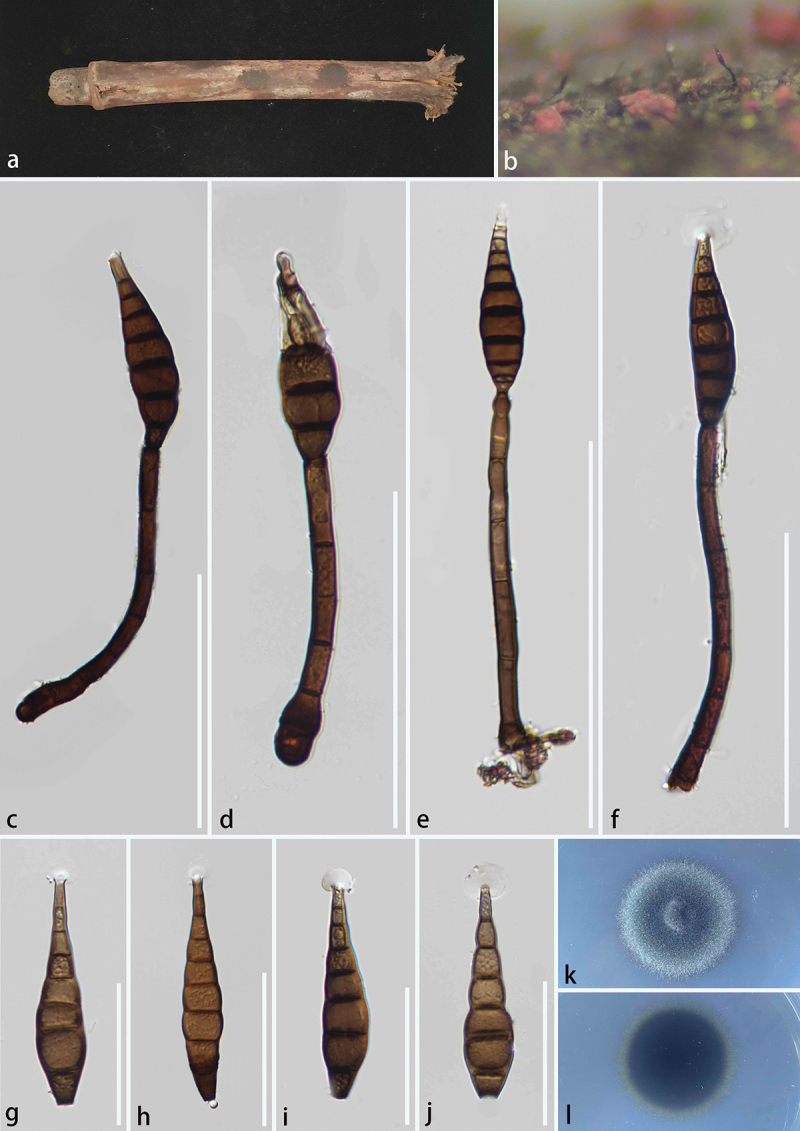
*Kirschsteiniothelia yadongensis* (HKAS 144543, holotype). (a) Host substrate. (b) Colonies on natural substrate. (c – f) Conidiophores and conidiogenous cells. (g – j) Conidia. (k, l) Culture on PDA. Scale bars: c – f = 100 μm, g – j = 50 μm.

***Kirschsteiniothelia yadongensis*** S.C. He, Q. Zhao & K.D. Hyde, sp. nov. Figure 3

*MycoBank number*: MB857899; *Facesoffungi number*: FoF17073.

*Etymology* – Referring to the collecting site of the type specimen, Yadong County, Shigatse City, Xizang Autonomous Region, China.

*Description* – *Saprobic* on decaying wood. **Asexual morph**: Hyphomycetous. *Colonies* immersed on the substrate, effuse, composed of septate, unbranched, brown to black. *Conidiophores* 92–146 × 4–8.5 μm ($\overset{ˉ}{x}$ = 120 × 7 μm, *n* = 20), macronematous, mononematous, erect, simple, straight or flexuous, unbranched, smooth, thick-walled, septate, pale brown to brown. *Conidiogenous cells* monoblastic, terminal, cylindrical, brown, terminal developing into conidia. *Conidia* 61–97 × 16–21 μm ($\overset{ˉ}{x}$ = 77 × 19 μm, *n* = 20), solitary, acrogenous, simple, obclavate, smooth, guttulate, thick-walled, 6–8-septate, brown to black brown, lighter apex with a hayline gelatinous sheath. **Sexual morph**: Not observed.

*Culture characteristics* – germinating within 24 h on PDA at 25 °C, reaching 2.5–3.0 cm after 20 days incubation, above and reverse both black with white margin, filamentous, raised, surface smooth, medium dense, no pigment.

*Host* – On unknown substrate.

*Known distribution* – China, Xizang Autonomous Region, Shigatse City, Yadong County.

*Material examined* – China, Xizang Autonomous Region, Shigatse City, Yadong County (27°63′N, 88°92′E, 3,707 m), on unknown wood of a deciduous tree, 29 July 2023, Shucheng He, ZYW119 (KUN-HKAS 144543, **holotype**) – ex-type, KUNCC24-18543.

*Notes* – Phylogenetically, *K. yadongensis* formed a monophyletic clade with *K. phoenicis* (92% ML/1.00 PP, Figure 1). *Kirschsteiniothelia phoenicis*, which is saprobic on *Phoenix paludosa* (Arecaceae) and was reported from Thailand, was described by Hyde et al. (2018) based on its sexual morphic characteristics and phylogenetic results (ITS, SSU, and LSU). Because *Kirschsteiniothelia phoenicis* only reports the sexual morph (Hyde et al. 2018), and our isolates only report the asexual morph, there is no morphological comparison. It is distinguishable *K. yadongensis* from *K. phoenicis* based on morphology (Hyde et al. 2018). However, *K. yadongensis* differs from *K. phoenicis* by 49/510 (9.6%, without gaps) ITS, 11/810 (1.3%, without gaps) LSU, and 13/805 (1.6%, without gaps) SSU differences in the nucleotides comparisons. Thus, we propose a new species *K. yadongensis* from Shigatse City, Xizang Autonomous Region, China.

**Astrosphaeriellaceae** Phook. & K.D. Hyde

**Figure 4.**
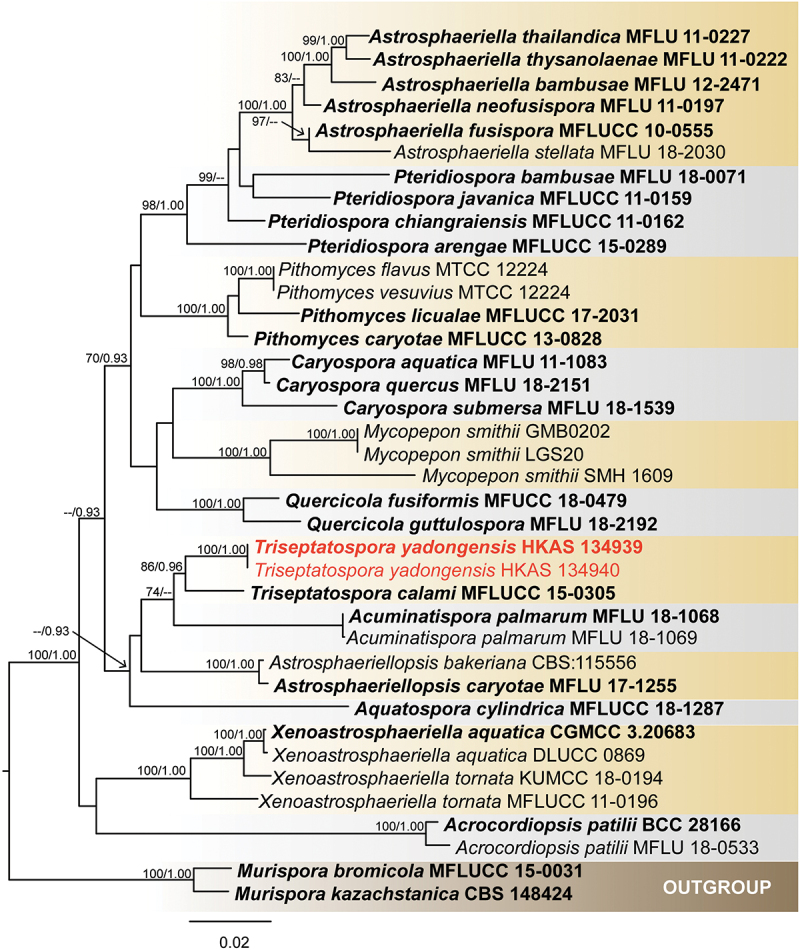
Maximum likelihood tree of Astrosphaeriellaceae from a three-locus dataset (SSU, LSU, and *tef*1). RAxML bootstrap support values (ML ≥ 70%) and Bayesian posterior probability (PP ≥ 0.90) are shown at the nodes (ML/PP). The scale bar indicates 0.03 changes per site. *Murispora kazachstanica* (CBS 148424) and *M. bromicola* (MFLUCC 15-0031) were selected as outgroup. The type strains are in bold while the newly generated sequences are indicated in red.

The SSU+LSU+*tef*1 dataset includes sequences from 38 samples representing 31 species in the GenBank database (Table 3). *Murispora kazachstanica* and *Murispora bromicola* are selected as outgroups following Crous et al. (2021). After comparison, the LSU, SSU, and *tef*1 were assembled (SSU = 1–908 bp; LSU = 909–1,689 bp; *tef*1 = 1,690–2,561 bp), and the phylogenetic trees of ML and BI were constructed. The phylogenetic analysis results showed that the total number of combined sequence sites was 2,561 including gaps. The matrix had 654 distinct alignment patterns, with 17.40% of undetermined characters or gaps. Estimated base frequencies were as follows: A = 0.244259, C = 0.245957, G = 0.280553, T = 0.229231; substitution rates: AC = 1.245609, AG = 3.886107, AT = 1.345370, CG = 1.060643, CT = 13.468140, GT = 1.000000; gamma distribution shape parameter α = 0.426349. The best-scoring RAxML tree had a final likelihood value of −11,241.554985. *Triseptatospora yadongensis* (HKAS 134939 and HKAS 134939) formed a distinct clade with *T. calami* with support value of (86% ML/0.96 PP, Figure 4).

***Triseptatospora*** Konta & K.D. Hyde. Mycosphere 14(1): 117 (2023)

*Triseptatospora* (Astrosphaeriellaceae, Pleosporales), a monotypic genus, was introduced by Konta et al. (2023) to accommodate the type species *Triseptatospora calami. Triseptatospora* was collected from *Calamus* sp. in the terrestrial habitat of Thailand. Morphologically, only the sexual morph has been reported. The genus is characterised by scattered to gregarious, superficial, raised, subglobose to dome-shaped, clustered, black, ostiolate ascomata, 6–8-spored, bitunicate, short pedicels asci, fusiform, 3-septate, hyaline, guttulate, mucilaginous sheath ascospores (Konta et al. 2023).

**Figure 5.**
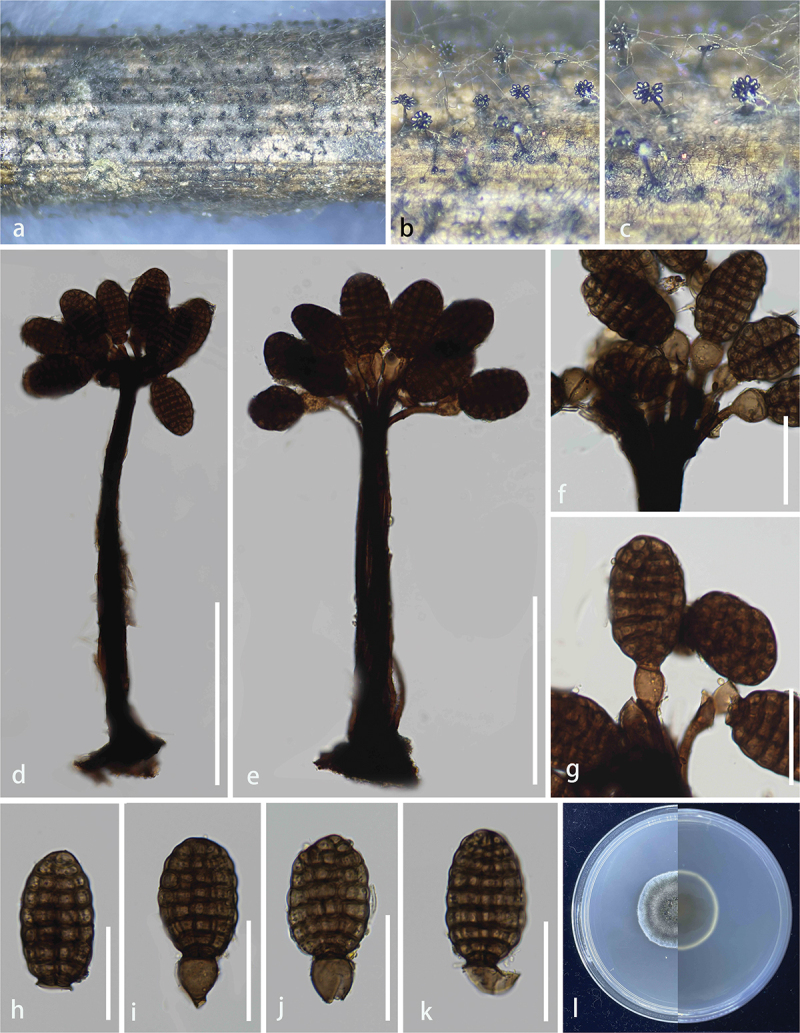
*Triseptatospora yadongensis* (HKAS 134939, holotype). (a) Host substrate. (b, c) Synnemata on the host surface. (d, e) Synnemata composed of conidiophores. (f, g) Conidiogenous cells. (h – k) Conidia. (l) Culture on PDA. Scale bars: d, e = 100 μm, f – k = 50 μm.

***Triseptatospora yadongensis*** S.C. He, Jayaward. & Q. Zhao, sp. nov. Figure 5

*MycoBank number*: MB 857900; *Facesoffungi number*: FoF17074.

*Etymology* – Referring to the collecting site of the type specimen, Yadong County, China.

*Description* – Saprobic on unknown wood. **Asexual morph**: Hyphomycetous. *Synnemata* 270–430 × 20–27 μm ($\overset{ˉ}{x}$ = 360 × 24 μm, *n* = 10), on host surface, synnematous, synnemata composed of conidiophores, erect, solitary, straight, terminal branched, closely compacted hyphae, with brown to dark brown stems, terminal developing into conidiogenous cells and conidia. *Conidiophores* 280–340 × 2.8–3.5 μm ($\overset{ˉ}{x}$ = 325 × 3 μm, *n* = 10), straight, unbranched, smooth, thick-walled, rough-walled, septate, brown, terminal developing into conidiogenous cells. *Conidiogenous cells* 14–28 × 10–17 μm ($\overset{ˉ}{x}$ = 19 × 13 μm, *n* = 10), monoblastic, integrated, terminal, determinate, connected at the base of conidia, brown. *Conidia* 55–71 × 32–50 μm ($\overset{ˉ}{x}$ = 63 × 40 μm, *n* = 20), dictyospores, solitary, acrogenous, simple, papillate, rugose, thick-walled, rough-walled, 4–6 transverse septa, 7–9 longitudinal septa, yellow-brown, constricted at the septa. **Sexual morph**: Not observed.

*Culture characteristics* – Conidia germinating within 20 h on PDA at 25 °C, reaching 2.5–3 cm after 20 days incubation, colony surface pale olivaceous-grey, reverse grey green, dense, circular, slightly raised, smooth, entire, hairy, wrinkled folded, pigmentation not produced on PDA.

*Host* – On unknown substrate.

*Known distribution* – China, Xizang Autonomous Region, Shigatse City, Yadong County.

*Material examined* – China, Xizang Autonomous Region, Shigatse City, Yadong County (27°23’N, 89°01′E, 1,687 m), 25 July 2023, Shucheng He, CT20 (KUN-HKAS 134939, **holotype**) – ex-type: KUNCC 24-17789. *ibid*. Shucheng He, CT24 (KUN-HKAS 134940, **paratype**) – ex-paratype: KUNCC24-17790.

*Notes* – The isolates formed a monophyletic clade with *T. calami* (Figure 4). Morphologically, our collections have distinct synnemata, which were first reported in Astrosphaeriellaceae (Liu et al. 2018; Wanasinghe et al. 2018; Dong et al. 2020; Crous et al. 2021; Luo et al. 2022; Konta et al. 2023). The isolates are similar to *Kostermansinda* in morphology, have synnematous and dictyospores, acrogenous, papillate conidia, however, the conidia of *Kostermansinda* have multiple (12–13 *vs.* 7–9) longitudinal septa (Boedijn 1960; Rifai 1968). The phylogenetic position of *Kostermansinda* has not been resolved as they lack DNA-based sequence data. Comparison of LSU, mtSSU, *rpb*2, and *tef*1 sequences in the NCBI database revealed that they belong to the orders Lecanorales, Monoblastiales, and Tubeufiales, respectively (Hosoya and Yano 2020). Species in *Triseptatospora* are known only by sexual morphs (Konta et al. 2023), the nucleotide differences of SSU and *tef*1 between our isolates and *Triseptatospora calami* are 3/793 (0.4%, without gaps) and 47/956 (5%, without gaps), respectively (Konta et al. 2023). Based on this evidence, following Chethana et al. (2021), we have retained our isolate in *Triseptatospora* and propose a new species, *T. yadongensis*, from Shigatse City, Xizang Autonomous Region, China.

**Dictyosporiaceae** Boonmee & K.D. Hyde

**Figure 6.**
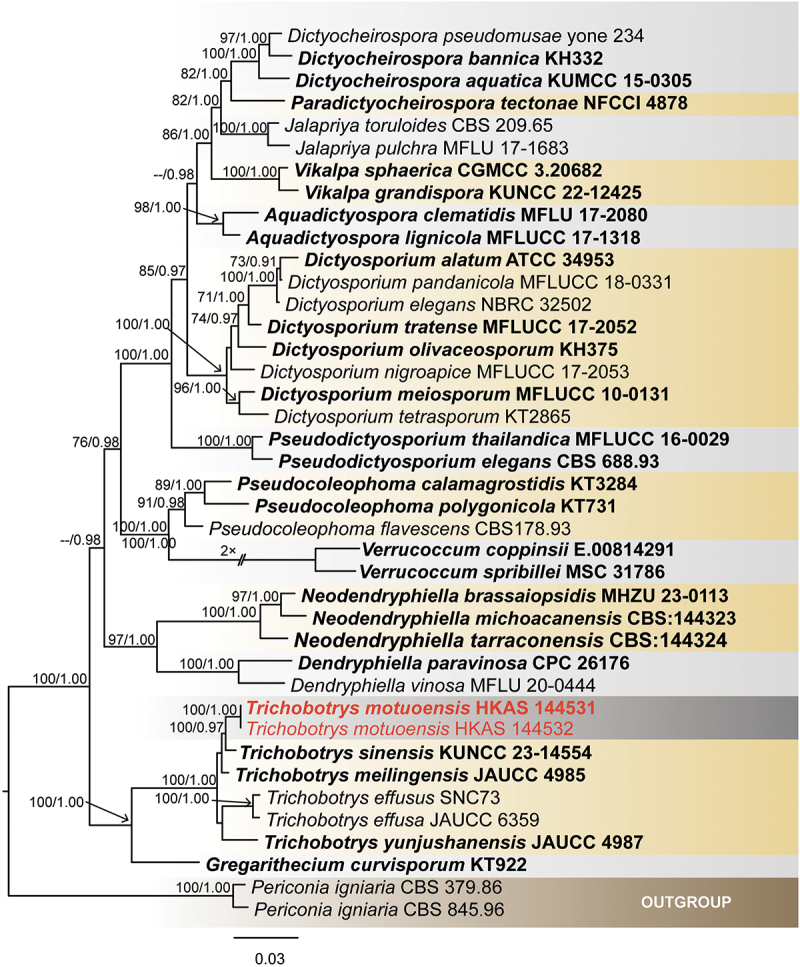
Maximum likelihood tree of Dictyosporiaceae reconstructed from a four-locus dataset (LSU, SSU, ITS, and *tef*1). RAxML bootstrap support values (ML ≥ 70%) and Bayesian posterior probability (PP ≥ 0.90) are shown at the nodes (ML/PP). The scale bar indicates 0.03 changes per site. *Periconia igniaria* (CBS 379.86 and CBS 845.96) was selected as outgroup. The type strains are in bold while the newly generated sequences are indicated in red.

The combined ITS, LSU, SSU, and *tef*1 datasets comprise 40 taxa, with *Periconia igniaria* (Su et al. 2023) as the outgroup taxa (Table 4). The dataset consisted of 3,967 total characters, including gaps (LSU: 1–826 bp, SSU: 827–1,751 bp, ITS: 1,752–2,271 bp, *tef*1: 2,272–3,099 bp). The matrix had 818 distinct alignment patterns, with 21.01% of undetermined characters or gaps. Estimated base frequencies were as follows: A = 0.243068, C = 0.241628, G = 0.269153, T = 0.246152; substitution rates: AC = 1.684401, AG = 3.847836, AT = 2.349320, CG = 0.793813, CT = 9.946496, GT = 1.000000; gamma distribution shape parameter α = 0.457026. The best-scoring RAxML tree with a final likelihood value of −15,213.255591 is presented in Figure 6. The tree topology obtained from ML analysis is similar to the one inferred from BI analysis. Based on the phylogenetic analyses of the combined ITS, LSU, and SSU sequence data, the novel species *Trichobotrys motuoensis* (HKAS 144531 and HKAS 144532) is closely related to *T. sinensis* with 100% ML/0.97 PP statistical support (Figure 6).

***Trichobotrys*** Penz. & Sacc., Malpighia 15(7–9): 245 (1902) [1901]

*Trichobotrys* was introduced by Penzig and Saccardo (1902) to accommodate the type species *Tr. pannosus*. Later, *Tr. pannosus* was treated as a synonym of *Tr. effusa* (Morgan-Jones et al. 1987; D’Souza and Bhat 2001). *Trichobotrys* has eight epithets in Index Fungorum (2025, accessed on 7 May 2025) and among them, only four species (*Tr. effusa*, *Tr. meilingensis*, *Tr. sinensis*, and *Tr. yunjushanensis*) have been molecularly confirmed (Phookamsak et al. 2024; Zhang et al. 2024). The genus is known solely by its asexual morph, which is characterised by solitary or clustered in a small group, scattered to gregarious, globose to subglobose, ostiolate, minute papilla conidiomata, dark brown to black, effuse colonies, macronematous, mononematous, brown, verruculose or echinulate conidiophores, smooth, fertile, often unciform lateral branches, with sterile, setiform apex, polyblastic, integrated, terminal or discrete, ellipsoidal, spherical or subspherical conidiogenous cells and catenated, spherical, brown, aseptate, verruculose or echinulate conidia (D’Souza and Bhat 2001; Phookamsak et al. 2024; Zhang et al. 2024). Species of *Trichobotrys* were reported in China and Java, from both aquatic and terrestrial habitats, where they are saprobic on Arecaceae and Poaceae (*Brachiaria mutica* and Bamboo) (D’Souza and Bhat 2001; Phookamsak et al. 2024; Zhang et al. 2024).

**Figure 7.**
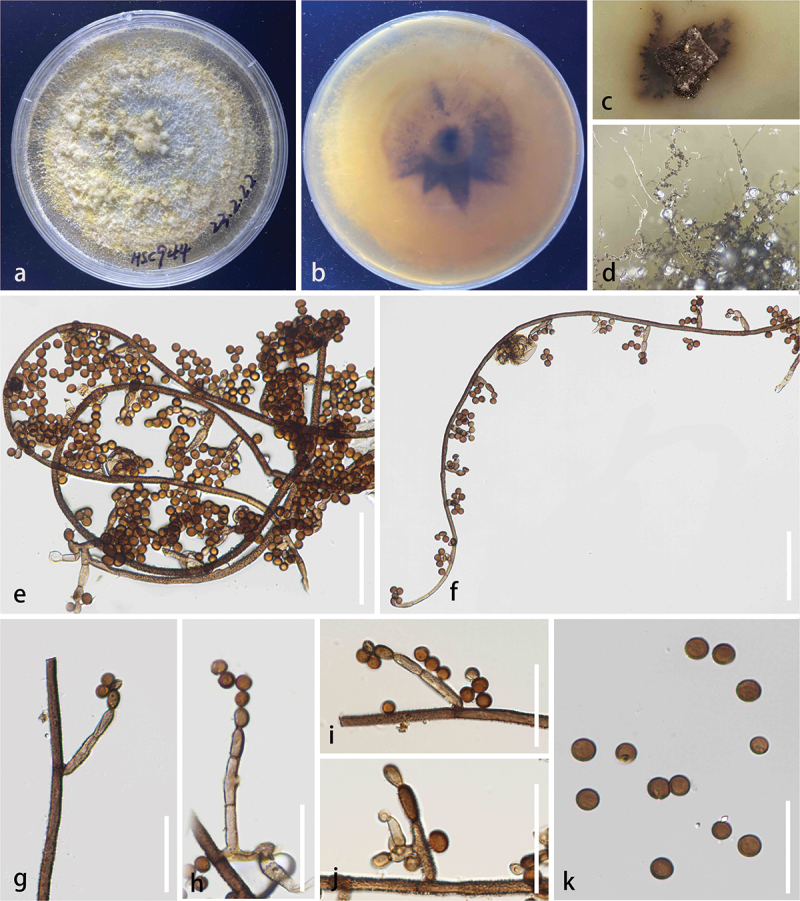
*Trichobotrys motuoensis* (HKAS 144531, holotype). (a, b) Culture on PDA. (c, d) Appearance of colonies on SNA. (e, f) Conidiophores. (g – j) Conidiogenous cells. (k) Conidia. Scale bars: e, f = 50 μm, g – k = 25 μm.

***Trichobotrys motuoensis*** S.C. He, Q. Zhao & K.D. Hyde, sp. nov. Figure 7

*MycoBank number*: MB857901; *Facesoffungi number*: FoF17075.

*Etymology* – Referring to the collecting site of the type specimen, Motuo County, China.

*Description* – **Asexual morph**: Hyphomycetous. *Colonies* on the Synthetic Low Nutrient Agar (SNA), effuse, branched, dark brown, hairy. *Conidiophores* 800–1,100 μm long, 3–5 μm wide ($\overset{ˉ}{x}$ = 4 μm, *n* = 20), dichotomously branched, mononematous, simple, flexuous, branched or occasionally loosely branched, verruculose, thick-walled, rough-walled, echinulate, septate, pale brown to brown. *Conidiogenous cells* 14–20 × 4–4.6 μm ($\overset{ˉ}{x}$ = 17 × 4 μm, *n* = 20), polyblastic, integrated, terminal, hyaline to pale brown, verruculose, thick-walled, denticulate. *Conidia* 5–6 × 5–6 μm ($\overset{ˉ}{x}$ = 5.5 × 5.5 μm, *n* = 30), catenate, acropleurogenous, solitary, simple, dry, verruculose, echinulate, globose, thin-walled, smooth-walled, aseptate, brown. **Sexual morph**: Not observed.

*Culture characteristics* – on PDA at 25 °C, reaching 6.0–6.2 cm after 45 days incubation, above colony pale yellow, reverse yellow edge with a black centre, irregular, raised, umbonate, surface rough, dense, no pigment.

*Host* – On unknown substrate.

*Known distribution* – China, Xizang Autonomous Region, Linzhi City, Motuo County.

*Material examined* – China, Xizang Autonomous Region, Linzhi City, Motuo County (29°25′N, 95°20′E, 792 m), on unidentified substrate, 6 July 2022, Shucheng He, HSC944 (KUN-HKAS 144531, **holotype**) – ex-type – KUNCC24-18551.

*Notes* – The phylogenetic tree (LSU, SSU, ITS, and *tef*1) showed that *Tr. motuoensis* was clustered with *Tr. sinensis* and *Tr. meilingensis* (Figure 6). Morphologically, *Tr. motuoensis* resembles *Tr. sinensis* and *Tr. meilingensis* in having acropleurogenous, verruculose, echinulate, globose conidia. However, *Tr. motuoensis* has larger conidia compared to *Tr. sinensis* (5.5–5.5 µm *vs*. 2.8–2 µm), and smaller conidia compared to *Tr. meilingensis* (5.5–5.5 µm *vs*. 7–13 µm) (Phookamsak et al. 2024; Zhang et al. 2024). *Trichobotrys sinensis* and *Tr. meilingensis* have both been found in aquatic environments in China. *Trichobotrys sinensis* is saprobic on *Brachiaria mutica* (Poaceae), while *Tr. meilingensis* is saprobic on decaying bamboo (Phookamsak et al. 2024; Zhang et al. 2024). The nucleotide differences for ITS, LSU, SSU, and *tef*1 between *Tr. motuoensis* and *Tr. sinensis* are 6/480 (1.2%, without gaps), 2/770 (0.3%, without gaps), 2/763 (0.3%, without gaps), and 15/870 (1.7%, without gaps), respectively. Similarly, the nucleotide differences for ITS, LSU, SSU, and *tef*1 between *Tr. motuoensis* and *Tr. meilingensis* are 6/501 (1.2%, without gaps), 2/800 (0.2%, without gaps), 3/752 (0.4%, without gaps), and 11/774 (1.4%, without gaps), respectively. Therefore, we introduce *Tr. motuoensis* as a new species, from Motuo County, Xizang Autonomous Region, China.

**Periconiaceae** Nann.

**Figure 8.**
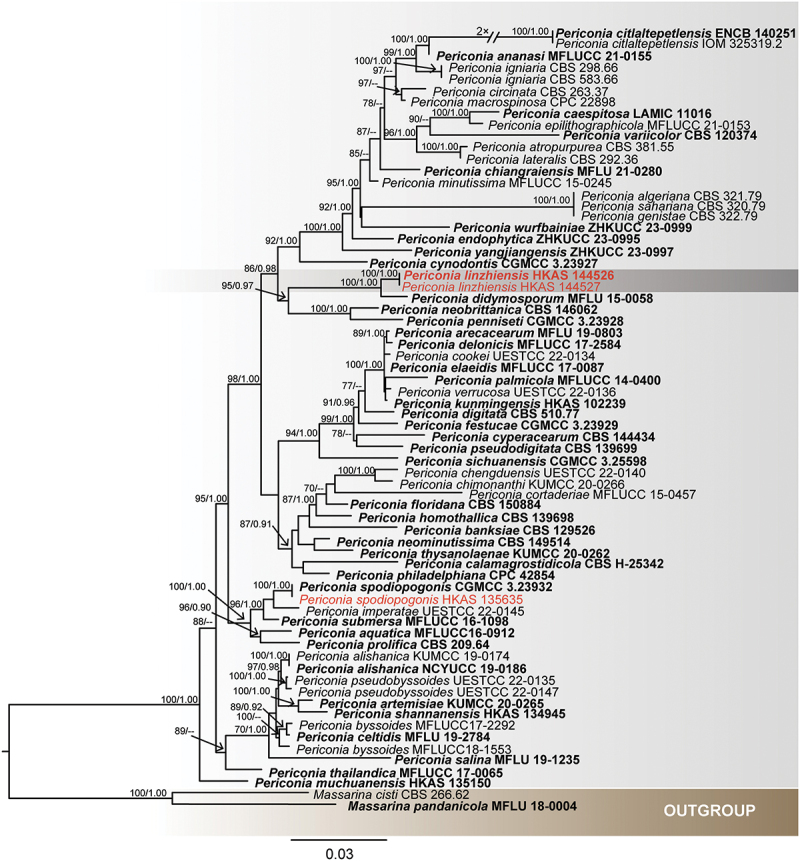
Maximum likelihood tree of *Periconia* reconstructed from a four-locus dataset (ITS, SSU, LSU, and *tef*1). RAxML bootstrap support values (ML ≥ 70%) and Bayesian posterior probability (PP ≥ 0.90) are shown at the nodes (ML/PP). The scale bar indicates 0.02 changes per site. *Massarina cisti* (CBS 266.62) and *M. pandanicola* (MFLU 18-0004) were selected as outgroup taxa. The type strains are in bold while the newly generated sequences are indicated in red.

The combined LSU, SSU, ITS, and *tef*1 dataset comprises 68 taxa, with *Massarina cisti* (Tanaka et al. 2015) and *Massarina pandanicola* (Tibpromma et al. 2018) designated as the outgroup taxon (Table 5). The dataset consists of 3,118 total characters including gaps (LSU: 1–823 bp, SSU: 824–1,770 bp, *tef*1: 1,771–2,618 bp, ITS: 2,619–3,118 bp). The matrix had 802 distinct alignment patterns, with 25.80% of undetermined characters or gaps. Estimated base frequencies were as follows: A = 0.236633, C = 0.256358, G = 0.270666, T = 0.236343; substitution rates: AC = 1.797398, AG = 2.963500, AT = 2.015171, CG = 1.254214, CT = 10.715473, GT = 1.000000; gamma distribution shape parameter α = 0.515216. The best-scoring RAxML tree had a final likelihood value of −15,110.354168. Both the ML and BI analyses yielded similar tree topologies, and only the tree inferred from ML analysis is shown (Figure 8). Our isolates (HKAS 144526 and HKAS 144527) formed a well-supported, distinct clade with *Periconia didymosporum* (100% ML/1.00 PP, Figure 8). In addition, we identify *P. spodiopogonis* as a new host record for China.

***Periconia*** Tode, Fung. mecklenb. sel. (Lüneburg) 2: 2 (1791)

*Periconia* (Periconiaceae, Pleosporales) was introduced by Tode (1791) with *P. lichenoides* as the type species. This genus primarily comprises species known for their asexual morphs, characterised by macronematous, mononematous conidiophores, and globose to ellipsoidal conidia (Phookamsak et al. 2019; Hongsanan et al. 2020; Liao et al. 2024). The sexual morph is characterised by immersed to erumpent, scattered or aggregated, subglobose to globose ascomata, hyaline, 8-spored, bitunicate, cylindrical, septate, guttulate, ascospores with an entire sheath (Tanaka et al. 2015; Yang et al. 2022; Su et al. 2023; Sun et al. 2025). *Periconia* species are mostly saprophytes and endophytes, though some are plant pathogens such as *P. circinata* and *P. macrospinosa* (Kolomiets et al. 2008; Sarkar et al. 2019) and are widely distributed in terrestrial habitats. The genus includes 234 records (Index Fungorum 2025, accessed on 7 May 2025). These fungi have a cosmopolitan distribution and inhabit various environments, including soil, decaying plant material, and living plant tissues. Notable species include *P. byssoides*, *P. digitate*, and *P. minutissima*, found on diverse hosts such as grasses, legumes, and daisies (Hyde et al. 2020; Crous et al. 2023).

**Figure 9.**
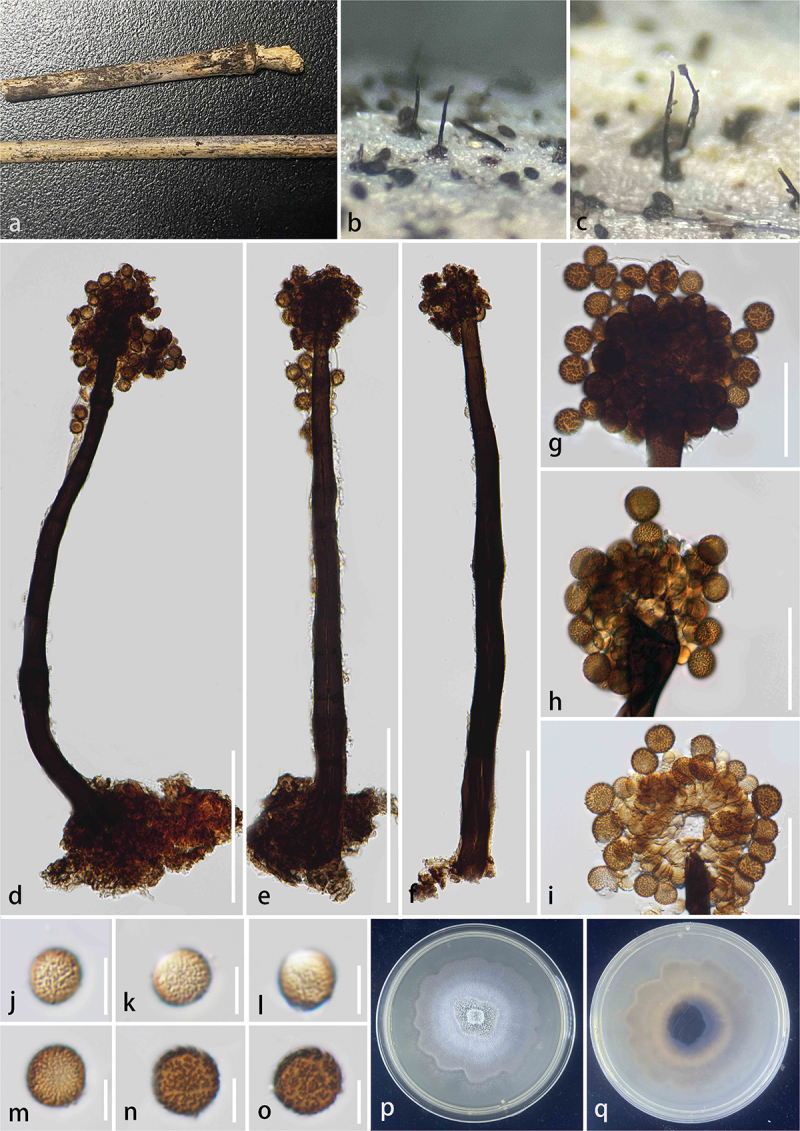
*Periconia linzhiensis* (HKAS 144526, holotype). (a) Host substrate. (b – c) Colonies on natural substrate. (d – f) Conidiophores. (g – i) Conidiogenous cells. (j – o) Conidia. (p, q) Culture on PDA. Scale bars: d – f = 100 μm, g – i = 50 μm, j – o = 10 μm.

***Periconia linzhiensis*** S.C. He, Q. Zhao & K.D. Hyde, sp. nov. Figure 9

*MycoBank number*: MB857902; *Facesoffungi number*: FoF17076.

*Etymology* – Referring to the collecting site of the type specimen, Linzhi City, Xizang Autonomous Region, China.

*Description* – *Saprobic* on dead bamboo. **Asexual morph**: Hyphomycetous. *Colonies* on the substrate, effuse, erect, brown to dark brown, hairy. *Conidiophores* 315–427 × 16–21 μm ($\overset{ˉ}{x}$ = 355 × 18 μm, *n* = 20), macronematous, mononematous, erect, simple, straight or flexuous, unbranched, thick and smooth-walled, septate, brown to dark brown. *Conidiogenous cells* polyblastic, terminal, subglobose, smooth to verruculose, hyaline to brown. *Conidia* 14–24 × 16–25 μm ($\overset{ˉ}{x}$ = 21 × 20 μm, *n* = 30), catenate, acrogenous, globose, simple, simple, verruculose, thick-walled, rough-walled, aseptate, pale brown when young; dark brown when mature. **Sexual morph**: Not observed.

*Culture characteristics* – Germinating within 24 h on PDA at 25 °C, reaching 5.5–6.0 cm after 30 days incubation, above colony brown black with grey centre; reverse brown, circular undulate, umbonate, surface rough, mycelia dense, no pigment.

*Host* – Bamboo.

*Known distribution* – China, Xizang Autonomous Region, Linzhi City, Motuo County.

*Material examined* – China, Xizang Autonomous Region, Linzhi City, Motuo County (29°35′N, 95°14′E, 1,730 m), on bamboo, 7 August 2023, Shucheng He, SBB30 (KUN-HKAS 144526, **holotype**) – ex-type, KUNCC25-19123.

*Notes* – *Periconia linzhiensis* formed a monophyletic clade with *P. didymosporum* with 100% ML/1.00 PP statistical support (Figure 8). *Periconia linzhiensis* and *P. didymosporum* were recorded from terrestrial environments and are saprophytic on Bamboo (Yang et al. 2022). However, *P. didymosporum* (= *Bambusistroma didymosporum*) was reported based on the sexual morph (Yang et al. 2022). The asexual morph of *Periconia linzhiensis* was reported. *Periconia linzhiensis* differed from *P. didymosporum* by 16/510 (3.2%, without gaps) ITS, 5/750 (0.6%, without gaps) LSU, 1/750 (0.1%, without gaps) SSU, and 7/810 (0.8%, without gaps) *tef*1 differences in the nucleotides. Thus, we propose *P. linzhiensis* as a new species.

***Periconia spodiopogonis*** Y.P. Chen & Z.H. Lu, Journal of Fungi 9: 18 (2023) Figure 10

**Figure 10.**
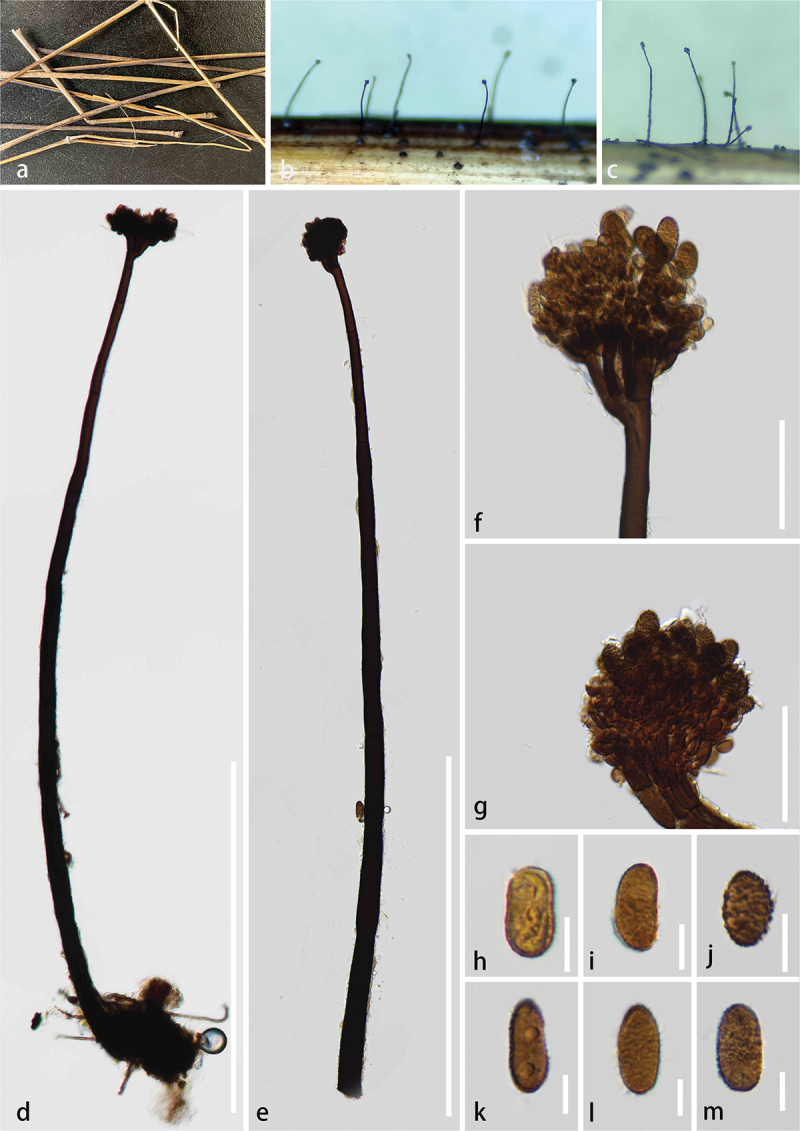
*Periconia spodiopogonis* (KHAS135635, new host record). (a) Host (Bamboo). (b, c) On the host surface. (d, e) Conidiophores. (f, g) Conidiogenous cells. (h – m) Conidia. Scale bars: d, e = 100 µm, f, g = 50 μm, h – m = 10 μm.

*MycoBank number*: MB847469; *Facesoffungi number*: FoF17077.

*Description* – *Saprobic* on dead bamboo. **Asexual morph**: Hyphomycetous. Colonies on the substrate, effuse, brown to dark brown, hairy. *Conidiophores* 430–720 × 16–27 μm ($\overset{ˉ}{x}$ = 590 × 22 μm, *n* = 20), macronematous, mononematous, erect, simple, straight or flexuous, branched at the apex, thick-walled, smooth-walled, septate, dark brown. *Conidiogenous cells* polyblastic, integrated, terminal, subglobose, smooth to verruculose, brown. *Conidia* 17–22 × 11–15 μm ($\overset{ˉ}{x}$ = 20 × 12.6 μm, *n* = 30), acrogenous, ellipsoidal to cylindrical, simple, verruculose, guttulate, thick-walled, rough-walled, aseptate, pale brown to dark brown. **Sexual morph**: Not observed.

*Material examined* – China, Xizang Autonomous Region, Linzhi City, Motuo County (29°17′N, 95°15′E, 1,550 m) on bamboo, 9 August 2023, Shucheng He, SBB04 (HKAS 135635).

*Known distribution* – China (Su et al. 2023).

*Known hosts* – *Spodiopogon* (Su et al. 2023), Bamboo (this study).

*Notes* – In the combined phylogenetic analyses (LSU, ITS, and SSU), our isolate was grouped together with *P. spodiopogonis*, having 100% ML/1.00 PP statistical support. *Periconia spodiopogonis* was initially described as a saprobe on *Spodiopogon* in China (Su et al. 2023). There were no differences in the ITS and SSU base sequences between our isolate and ex-type strain of *P. spodiopogonis*. Morphologically, our isolate (Figure 10) shares similar characteristics with *P. spodiopogonis* in conidiophores (590 × 22 *vs*. 595 × 15 μm) that are appressed to the substrate, macronematous, mononematous, erect, straight or flexuous and the *conidia* (20 × 12.6 *vs*. 20 × 12.5 μm) are acrogenous, ellipsoidal to cylindrical, verruculose, guttulate and aseptate (Su et al. 2023). Thus, based on phylogenetic analysis and morphological characteristics, we identified our isolate as *P. spodiopogonis*, which is a new host record in China.

**Tetraplosphaeriaceae** Kaz. Tanaka & K. Hiray

**Figure 11.**
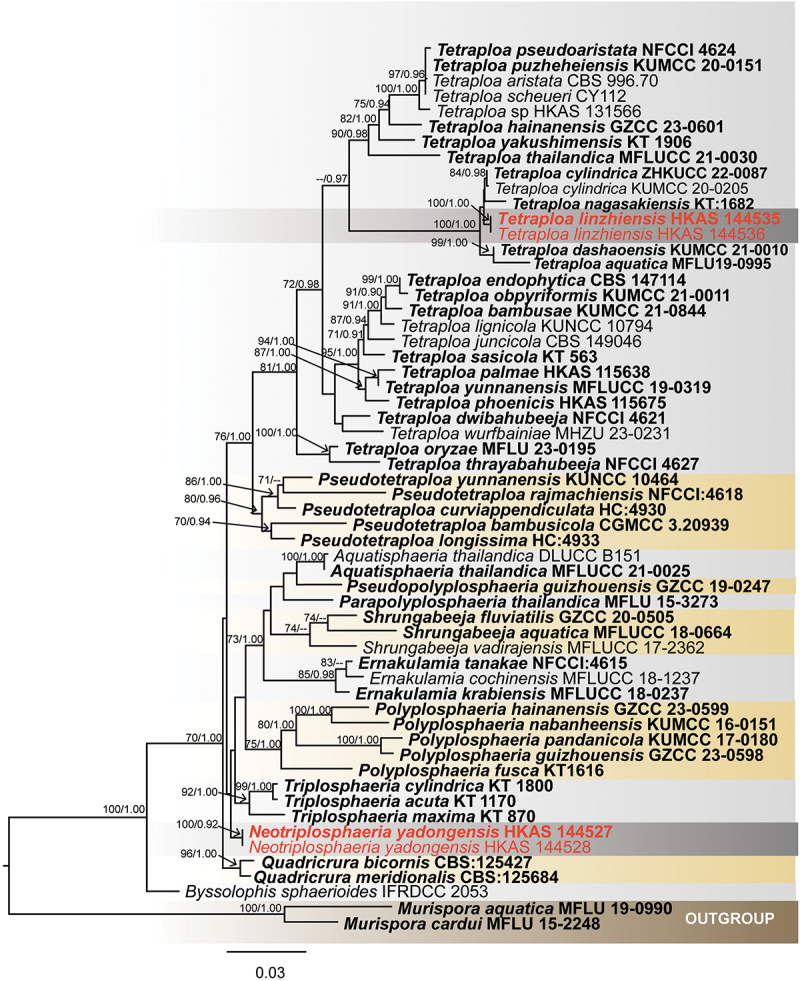
Maximum likelihood tree of Tetraplosphaeriaceae reconstructed from a three-locus dataset (LSU, SSU, and ITS). RAxML bootstrap support values (ML ≥ 70%) and Bayesian posterior probability (PP ≥ 0.90) are shown at the nodes (ML/PP). The scale bar indicates 0.03 changes per site. *Murispora aquatica* (MFLU 19-0990) and *Murispora cardui* (MFLU 15-2248) were selected as outgroup taxa. The type strains are in bold while the newly generated sequences are indicated in red.

In order to clarify the taxonomic status of four taxa, based on these four gene fragments, 58 fungal sequences of Tetraplosphaeriaceae were downloaded from the GenBank databases (Table 6), with *Murispora aquatica* and *Murispora cardui* as the outgroup (Bao et al. 2019). According to the LSU-SSU-ITS sequence data, the three genes were spliced (LSU = 1–821 bp; SSU = 822–1,747 bp; ITS = 1,748–2,289 bp). The total number of combined sequence sites is 2,289 including gaps. The matrix had 586 distinct alignment patterns, with 17.18% of undetermined characters or gaps. Estimated base frequencies were as follows: A = 0.247845, C = 0.241156, G = 0.282371, T = 0.228628; substitution rates: AC = 4.006945, AG = 4.409444, AT = 2.264925, CG = 1.075482, CT = 11.088939, GT = 1.000000; gamma distribution shape parameter α = 0.563830. The best-scoring RAxML tree had a final likelihood value of −11,459.593875. The ML tree topology was similar to the one inferred from BI analysis. The ML tree topology was similar to the one inferred from BI analysis. In the phylogenetic tree, each genus formed a separate clade, *Neotriplosphaeria yadongensis* forming a monophyletic clade in Tetraplosphaeriaceae, our isolates formed a monophyletic clade with *Tetraploa nagasakiensis* (Figure 11). Tetraplosphaeriaceae was established by Tanaka et al. (2009) to accommodate *Polyplosphaeria*, *Pseudotetraploa*, *Quadricrura*, *Tetraplosphaeria* (the type genus), and *Triplosphaeria*. It is characterised by massarina-like asexual morphs, immersed to superficial, glabrous, brown, cylindrical to clavate ascomata, and fusiform to cylindrical, narrowly, 8-spored asci, septate, hyaline to pale brown, with appendage-like sheath ascospores (Tanaka et al. 2009; Tian et al. 2024). The anamorphs of Tetraplosphaeriaceae are tetraploa-like hyphomycetes, micronematous, macronematous, erect, septate conidiophores, monoblastic, terminal conidiogenous cells, cylindrical to obpyriform, comprising 3–8 columns, verrucose, with 2–8-setose conidia (Tanaka et al. 2009). Based on morphological and molecular evidence (ITS, LSU, and SSU), Ariyawansa et al. (2015) introduced *Shrungabeeja* to Tetraplosphaeriaceae. Subsequently, *Aquatisphaeria*, *Byssolophis*, and *Ernakulamia* were introduced into the Tetraplosphaeriaceae (Delgado et al. 2017; Pem et al. 2019; Li et al. 2021). Recently, Zhang et al. (2023) introduced a new genus, *Pseudopolyplospha*, into Tetraplosphaeriaceae, which now consists of 10 genera (Zhang et al. 2023; Hyde et al. 2024b).

***Neotriplosphaeria*** S.C. He, Q. Zhao & K.D. Hyde, gen. nov.

*MycoBank number*: MB857903; *Facesoffungi number*: FoF17078.

*Etymology* – Named after its conidial similarity to *Triplosphaeria*.

*Description* – *Saprobic* on dead stems of bamboo. **Asexual morph**: *Colonies* effuse, medium dense, hairy, black. *Conidiophores* absent. *Conidiogenous cells* mononematous, monoblastic, integrated, terminal or intercalary, determinate, and cylindrical. *Conidia* solitary, septate, brown to dark brown, ovoid to obclavate, consisting of 2–4 columns of cells, multi-septate in each column, smooth, thick-walled, mostly with 2 to 4 apical appendages. *Appendages* cylindrical, solitary, unbranched, guttulate, septate, wide at the base, pale brown to brown, multi-septate, straight or flexuous, smooth-walled.

**Figure 12.**
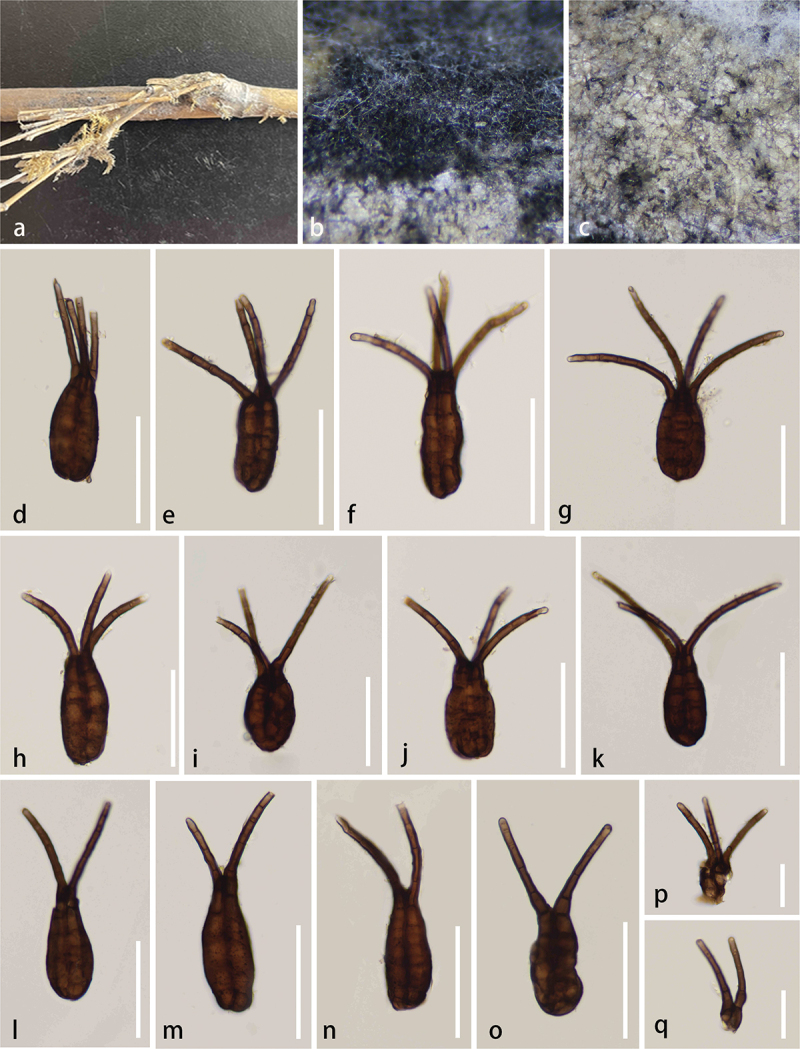
*Neotriplosphaeria yadongensis* (HKAS 144527, holotype). (a) Host substrate. (b, c) Appearance of colonies on natural substrate. (d – o) Conidia. (p, q) Appendages. Scale bars: d – o = 50 μm, p, q = 25 μm.

***Neotriplosphaeria*** ***yadongensis*** S.C. He, Q. Zhao & K.D. Hyde, sp. nov. Figure 12

*MycoBank number*: MB857904; *Facesoffungi number*: FoF17079.

*Etymology* – Refers to the collecting site of the type specimen, Yadong County, China.

*Description* – *Saprobic* on dead stems of bamboo. **Asexual morph**: *Colonies* effuse, medium dense, hairy, black. *Conidiophores* absent. *Conidiogenous cells* mononematous, monoblastic, integrated, terminal or intercalary, determinate, cylindrical. *Conidia* 42–60 × 18–27 μm ($\overset{ˉ}{x}$ = 49 × 22 μm, *n* = 30), solitary, septate, brown to dark brown, ovoid to obclavate, consisting of 2–4 columns of cells, 6–9-septate in each column, smooth, thick-walled, mostly with 2–4 apical appendages. *Appendages* 33–74 × 3–4 μm ($\overset{ˉ}{x}$ = 55 × 3.5 μm, *n* = 30), cylindrical, solitary, unbranched, guttulate, 4–6-septate, wide at the base, pale brown to brown, straight or flexuous, smooth-walled. **Sexual morph**: Not observed.

*Host* – Bamboo.

*Known distribution* – China, Xizang Autonomous Region, Shigatse City, Yadong County.

*Material examined* – China, Xizang Autonomous Region, Shigatse City, Yadong County (27°13′N, 80°00′E, 1,824 m), on bamboo, 24 July 2023, Shucheng He, CT10 (KUN-HKAS 144527, **holotype**).

*Notes* – *Neotriplosphaeria yadongensis* formed a distinct clade within Tetraplosphaeriaceae. Its morphology fits well with the generic concept of Tetraplosphaeriaceae with cylindrical to obpyriform, comprising 3–8 columns, verrucose, with 2–8 appendages conidia (Tanaka et al. 2009). Morphologically, the conidia of *Neotriplosphaeria* consist of 2–4 columns of cells with 2–4 apical appendages, while the conidia of *Triplosphaeria* consist of only three columns of cells with three apical appendages (Tanaka et al. 2009). *Neotriplosphaeria* has ovoid to obclavate conidia with 2–4 appendages of similar length, while *Quadricrura* has globose to subglobose conidia with long appendages that are usually 1–2 appendages, and short that are 4–5 appendages (Tanaka et al. 2009). Based on phylogenetic and morphological analyses, we propose a new genus, *Neotriplosphaeria*, within Tetraplosphaeriaceae to accommodate the new species *N. yadongensis* from Yadong County, Xizang Autonomous Region, China.

***Tetraploa*** Berk. & Broome, Ann. Mag. nat. Hist., Ser. 2 5: 459 (1850)

*Tetraploa* was introduced by Berkeley and Broome (1850) and was later assigned as the type genus of Tetraplosphaeriaceae (Tanaka et al. 2009). *Tetraploa* is the largest genus in Tetraplosphaeriaceae (Tang et al. 2023; Zhang et al. 2023), with 44 epithets currently listed in Index Fungorum (accessed on 25 August 2024). The sexual morph is characterised by immersed to erumpent, globose to subglobose, glabrous ascomata, fissitunicate, cylindrical to clavate, short-stalked asci with eight ascospores, and narrowly fusiform, hyaline, smooth ascospores with a narrow mucilaginous appendage-like sheath (Tanaka et al. 2009). The main characteristics of the asexual morph are cylindrical, brown, verrucose, aseptate conidia with four setose appendages at the apex (Tanaka et al. 2009; Tang et al. 2023; Zhang et al. 2023). Species of *Tetraploa* were commonly found as endophytes and saprobes on plant leaves and stems in both terrestrial and aquatic environments (Tanaka et al. 2009; Ariyawansa et al. 2015; Delgado et al. 2017; Pem et al. 2019; Hongsanan et al. 2020). They were documented on bamboo, other herbaceous plants, and in decaying wood and soil (Dong et al. 2020; Li et al. 2021). Notably, *Te. aristata* was reported as a pathogen affecting a variety of plants and humans (Markham et al. 1990; Hyde et al. 2013).

**Figure 13.**
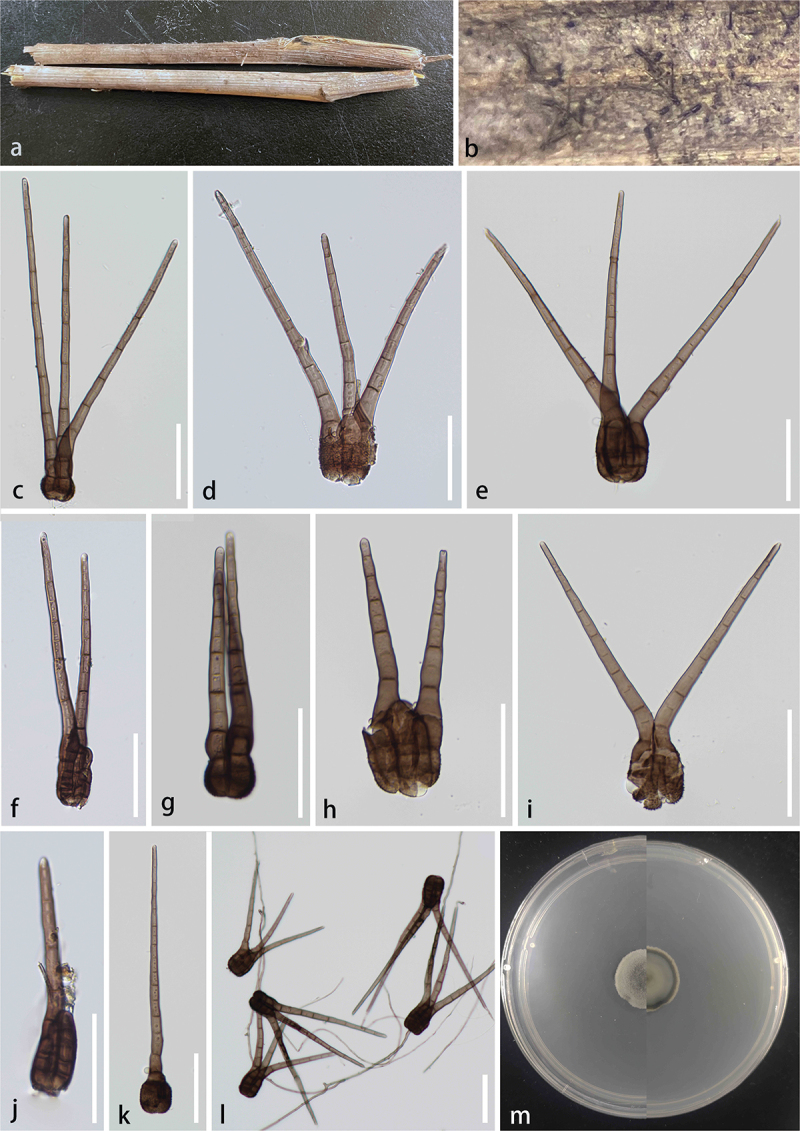
*Tetraploa linzhiensis* (HKAS 144535, holotype). (a) Host substrate. (b) Appearance of colonies on natural substrate. (c – i) Conidia. (j – l) Appendages. (m) Culture on PDA. Scale bars: c – l = 50 μm.

***Tetraploa*** ***linzhiensis*** S.C. He, Q. Zhao & K.D. Hyde, sp. nov. Figure 13

*MycoBank number*: MB857905; *Facesoffungi number*: FoF17080.

*Etymology* – Referring to the collecting site of the type specimen, Linzhi city, China.

*Description* – *Saprobic* on dead stems of *Elsholtzia* sp. **Asexual morph**: Hyphomycetous *Colonies* effuse, sparse, black. *Conidiophores* absent. *Conidiogenous cells* monoblastic, integrated, determinate. *Conidia* 25–45 × 19–30 μm ($\overset{ˉ}{x}$ = 39 × 23 μm, *n* = 30), solitary, cylindrical to obovate, septate, brown to dark brown, consisting of 1–3 columns of cells, 3–6-septate in each column, verruculose, thick-walled, rough-walled, with 1–3 apical appendages. *Appendages* 180–320 × 1–1.2 μm ($\overset{ˉ}{x}$ = 260 × 1.1 μm, *n* = 30), cylindrical, solitary, unbranched, wide at the base, pale brown to brown, hyaline at the apex, 6–9-septate, straight, guttulate, smooth-walled, long appendages, short appendages 102–188 × 7–9 μm ($\overset{ˉ}{x}$ = 137 × 8 μm, *n* = 30). **Sexual morph**: Not observed.

*Culture characteristics* – Germinating within 24 h on PDA at 25 °C, reaching 1.8–2 cm after 15 days incubation, above colony grey, reverse dark grey with black edge, entire, undulate, umbonate, surface smooth, mycelia dense, no pigment.

*Host* – *Elsholtzia* sp. (Lamiaceae).

*Known distribution* – China, Xizang Autonomous Region, Linzhi City, Chayu County.

*Material examined* – China, Xizang Autonomous Region, Linzhi City, Chayu County (28°54′N, 96°98′E, 1,878 m), on *Elsholtzia* sp., 13 August 2023, Shucheng He, ZYW49 (KUN-HKAS 144535, **holotype**) – ex-type culture in KUNCC24-18547.

*Notes* – The phylogenetic tree showed *Te. linzhiensis* was clustered with *Te. cylindrica* and *Te. nagasakiensis* with 100% ML and 1.00 PP support (Figure 11). Morphologically, *Te. linzhiensis* resembles *Te. cylindrica* and *Te. nagasakiensis* in having cylindrical to obovate, brown, multi columns of cells, verruculose, thick-walled, rough-walled conidia (Tanaka et al. 2009; Hyde et al. 2013). However, *Te. linzhiensis* mostly consists of three columns of cells and three appendages conidia, while *Te. cylindrica* and *Te. nagasakiensis* mostly consist of four columns of cells and four appendages conidia (Tanaka et al. 2009; Hyde et al. 2013). *Tetraploa cylindrica* and *Te. nagasakiensis* are both saprobic on Poaceae (*Sasa kurilensis* and bamboo) in Japan (Tanaka et al. 2009). The nucleotide differences for ITS, LSU, and SSU between *Te. linzhiensis* and *Te. cylindrica* are 10/505 (2%, without gaps), 3/748 (0.4%, without gaps), and 3/755 (0.4%, without gaps), respectively. Similarly, the nucleotide differences for ITS, LSU, and SSU between *Te. linzhiensis* and *Te. nagasakiensis* are 18/490 (3.6%), 2/732 (0.3%), and 3/782 (0.4%), respectively. Therefore, based on phylogenetic and morphological analyses, we propose a new species, *Te. linzhiensis*, from Linzhi City, Xizang Autonomous Region, China.

### Torulaceae Corda

**Figure 14.**
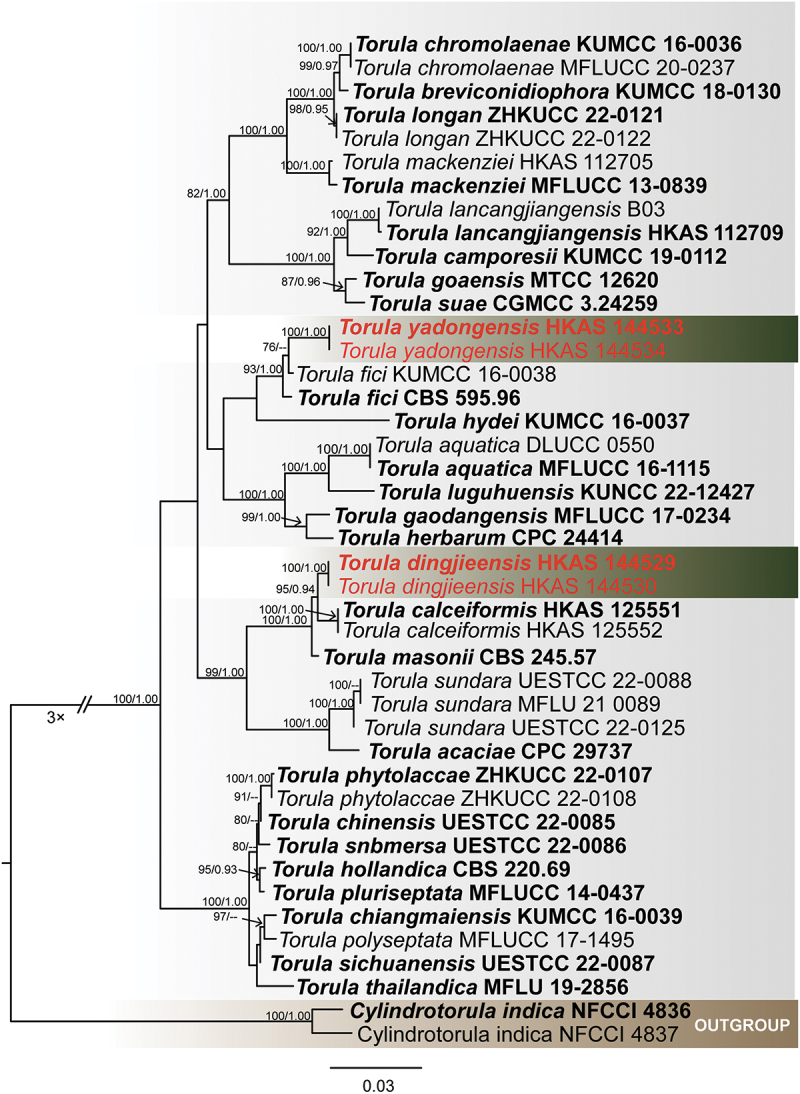
Maximum likelihood tree of *Torula* reconstructed from a five-locus dataset (LSU, SSU, ITS, *tef*1, and *rpb*2). RAxML bootstrap support values (ML ≥ 70%) and Bayesian posterior probability (PP ≥ 0.90) are shown at the nodes (ML/PP). The scale bar indicates 0.03 changes per site. *Cylindrotorula indica* (NFCCI 4836 and NFCCI 4837) was selected as outgroup. The type strains are in bold while the newly generated sequences are indicated in red.

The combined ITS, LSU, SSU, *tef*1, and *rpb*2 dataset comprised 43 taxa, with *Cylindrotorula indica* (Boonmee et al. 2021) as the outgroup taxa (Table 7). The dataset consisted of 3,920 total characters, including gaps (LSU: 1–806 bp, SSU: 807–1,715 bp, ITS: 1,716–2,214 bp, *tef*1: 2,215–3,080 bp, *rpb*2: 3,081–3,920 bp). The matrix had 842 distinct alignment patterns, with 26.55% of undetermined characters or gaps. Estimated base frequencies were as follows: A = 0.247197, C = 0.259213, G = 0.270214, T = 0.223376; substitution rates: AC = 2.116465, AG = 4.272528, AT = 1.684889, CG = 1.309028, CT = 9.872550, GT = 1.000000; gamma distribution shape parameter α = 0.521756. The best-scoring RAxML tree with a final likelihood value of −14,127.258641 is presented in Figure 14. The tree topology obtained from ML analysis is similar to the one inferred from BI analysis. The best-scoring RAxML tree is presented in (Figure 14). Our specimens *Torula dingjieensis* (HKAS 144529 & HKAS 144530) and *T. yadongensis* (HKAS 144533 & HKAS 144534) formed distinct monophyletic clades with *T. fici* and *T. calceiformis* with support value of 87% ML/0.92 PP and 95% ML/0.97 PP, indicating they are closely related.

***Torula*** Pers., Ann. Bot. (Usteri) 15: 25 (1795)

*Torula* was introduced by Persoon (1795) with the type species of *To. herbarum*. Most species of *Torula* were found in terrestrial and freshwater habitats on submerged wood or dead branches of Asteraceae, Cyperaceae, and Ranunculaceae in Asia, Europe, North America, and other regions (Crous et al. 2015; Hyde et al. 2017; Hongsanan et al. 2020; Jayawardena et al. 2022). *Torula* is the biggest genus in Torulaceae with 542 epithets recorded in Index Fungorum (24 November 2024). However, only 30 species were molecularly confirmed (Hyde et al. 2023; Tian et al. 2023; Wang et al. 2023a). *Torula* was documented solely in its asexual morph (Jayawardena et al. 2022; He et al. 2024b).

**Figure 15.**
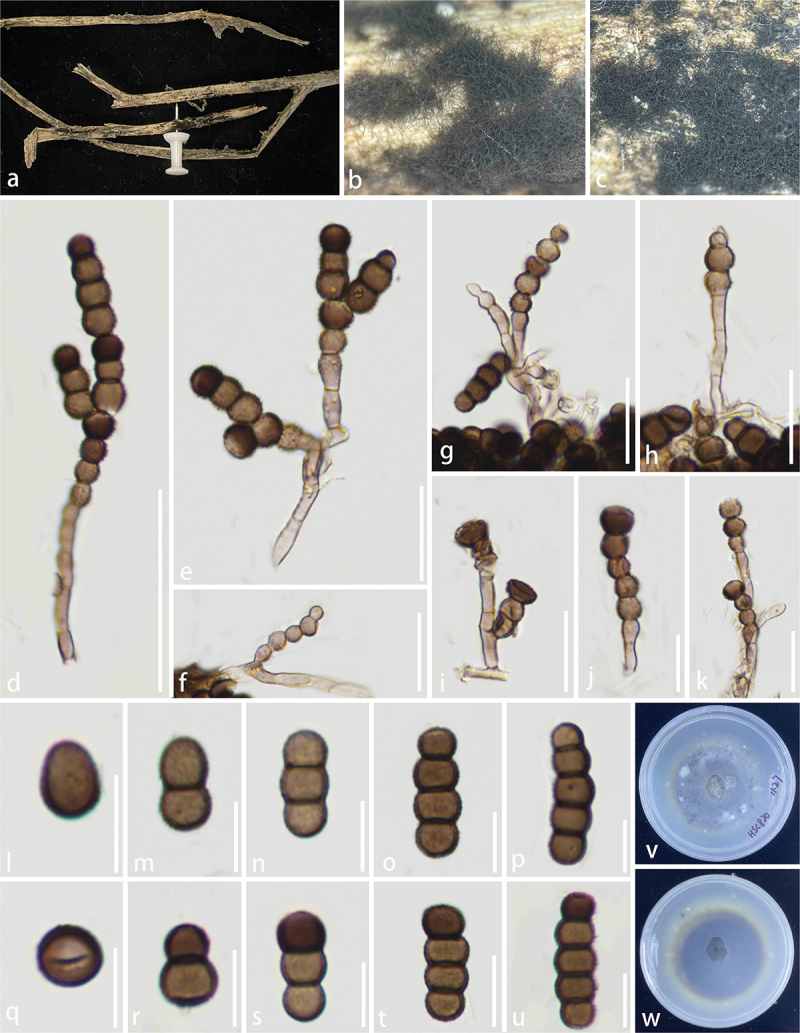
*Torula dingjieensis* (HKAS 144529, holotype). (a) Host substrate. (b, c) Appearance of colonies on natural substrate. (d, e) Conidiophores with conidiogenous cells. (f – k) Conidiogenous cells and conidia. (l – u) Conidia. (v, w) Culture on PDA. Scale bars: d = 50 μm, e – k, t, u = 20 μm, l – s = 10 μm.

***Torula dingjieensis*** S.C. He, Q. Zhao & K.D. Hyde, sp. nov. Figure 15

*MycoBank number*: MB857906; *Facesoffungi number*: FoF17081.

*Etymology* – Referring to the collecting site of the type specimen, Dingjie County, China.

*Description* – *Saprobic* on dead stems of *Ageratina adenophora*. **Asexual morph**: *Colonies* effuse, medium dense, black. *Conidiophores* semi-macronematous, mononematous, straight or flexuous, unbranched or occasionally loosely branched, verruculose, thick-walled, rough-walled, doliiform to subcylindrical, septate consisting of 1–4 cells or reduced to conidiogenous cells, pale brown to brown. *Conidiogenous cells* polyblastic, terminal, pale brown to brown, verruculose, thick-walled, doliiform to cupulate. *Conidia* 11–29 × 6–22 μm ($\overset{ˉ}{x}$ = 20 × 9 μm, *n* = 30), phragmospores, solitary, simple, dry, acrogenous, monilioid, verruculose, thick-walled, rough-walled, 0–4-septate, brown to dark brown. **Sexual morph**: Not observed.

*Culture characteristics* – germinating within 24 h on PDA at 25 °C, reaching 5.8–6.2 cm after 25 days incubation, above colony brown, reverse dark brown, both above and reverse with pale yellow edge, entire, undulate, umbonate, surface rough, mycelia dense, no pigment.

*Host* – *Ageratina adenophora* (Asteraceae).

*Known distribution* – China, Xizang Autonomous Region, Shigatse City, Dingjie County.

*Material examined* – China, Xizang Autonomous Region, Shigatse City, Dingjie County (27°54′N, 87°22′E, 2,240 m), on *Ageratina adenophora*, 4 July 2022, Shucheng He, HSC820 (KUN-HKAS 144529, **holotype**) – ex-type, KUNCC23-17478.

*Notes* – The phylogenetic tree showed that our isolates (HKAS 144529 and HKAS 144530) *To. dingjieensis* clustered with *To. calceiformis* (Figure 14). *Torula calceiformis* is saprobic on unidentified wood form Guizhou, China (Hyde et al. 2023). Our isolates (HKAS 144529 and HKAS 144530) were collected from *Ageratina adenophora* in Shigatse, Xizang Autonomous Region, China. Morphologically, *To. dingjieensis* resembles *To. calceiformis* in having semi-macronematous, mononematous, verruculose conidiophores, phragmospores, and monilioid, verruculose, multi-septate conidia. Compared to *To. dingjieensis*, *To. calceiformis* has conidia with additional septa (0–8-septate *vs*. 0–4-septate) and longer conidia (L/W ratio: 3 *vs*. 2.2) (Hyde et al. 2023). The nucleotide differences for ITS, LSU, SSU, *tef*1, and *rpb*2 between *To. dingjieensis* and *To. calceiformis* are 7/480 (1.5%, without gaps), 3/750 (0.4%, without gaps), 2/807 (0.2%, without gaps), 5/710 (0.7%, without gaps), and 21/885 (2.4%, without gaps), respectively (Hyde et al. 2023). Therefore, based on phylogenetic and morphological analyses, we propose a new species, *To. dingjieensis*, from Shigatse City, Xizang Autonomous Region, China.

**Figure 16.**
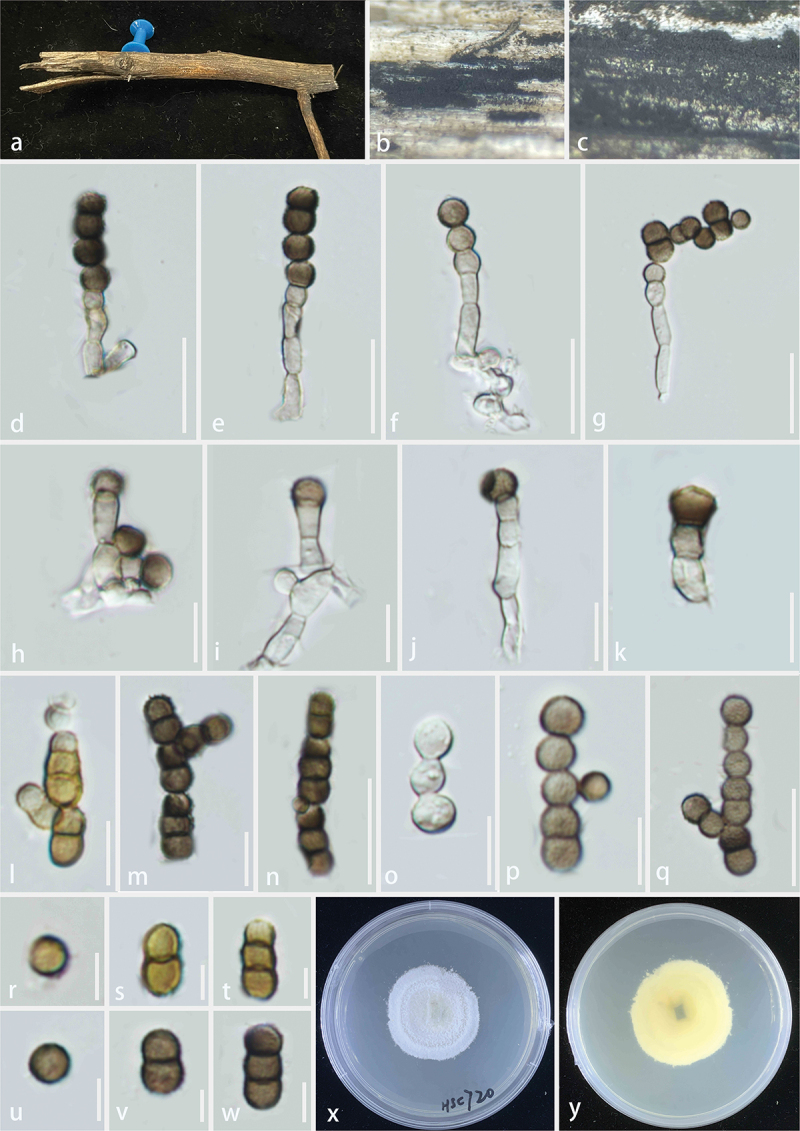
*Torula yadongensis* (HKAS 144533, holotype). (a) Host substrate. (b, c) Appearance of colonies on natural substrate. (d – g) Conidiophores. (h – k) Conidiogenous cells and conidia. (l – q) Branched chains of conidia. (r – w) Conidia. (x, y) Culture on PDA. Scale bars: d – g, n, q = 20 μm, h – m, o, p = 10 μm, r – w = 5 μm.

***Torula yadongensis*** S.C. He, Q. Zhao & K.D. Hyde, sp. nov. Figure 16

*MycoBank number*: MB857907; *Facesoffungi number*: FoF17082.

*Etymology* – Referring to the collecting site of the type specimen, Yadong County, China.

*Description* – *Saprobic* on dead stems of *Artemisia* sp. **Asexual morph**: *Colonies* effuse on nature substrate, medium dense, scattered, velutinous, black. *Conidiophores* macronematous, mononematous, straight or flexuous, branched of conidia chain, verruculose, thick-walled, rough-walled, doliiform to subcylindrical, septate, terminal reduced to conidiogenous cells, conidia chain hyaline or golden to dark brown. *Conidiogenous cells* monoblastic, terminal, pale brown to dark brown, verruculose, thick-walled, rough-walled, doliiform to cupulate. *Conidia* 8.4–14.6 × 4.3–6.2 μm ($\overset{ˉ}{x}$ = 12 × 6 μm, *n* = 30), didymospores, solitary, simple, pleuroacrogenous, monilioid, verruculose, thick-walled, rough-walled, 0–2-septate, golden to dark brown. **Sexual morph**: Not observed.

*Culture characteristics* – Germinating within 24 h on PDA at 25 °C, reaching 3.8–4.0 cm after 20 days incubation, both above and reverse white, entire, flat, surface smooth, mycelia dense, no pigment.

*Host* – *Artemisia* sp. (Asteraceae).

*Known distribution* – China, Xizang Autonomous Region, Shigatse City, Yadong County.

*Material examined* – China, Xizang Autonomous Region, Shigatse City, Yadong County (27°29′N, 88°54′E, 2,960 m), on *Artemisia* sp., 3 July 2022, Shucheng He, HSC720 (KUN-HKAS 144533, **holotype**) – ex-type KUNCC23-17229.

*Notes* – The new species was isolated from *Artemisia* sp. in Yadong County, China. Phylogenetic analyses (LSU-SSU-ITS-*tef*1-*rpb*2) showed *To. yadongensis* is closely related to *To. fici*, which was collected from leaves of *Ficus religiosa* (Moraceae) in Cuba. The phylogenetic tree showed *To. yadongensis* and *To. fici* were close relatives with 87% ML/0.92 PP support (Figure 14). *Torula yadongensis* resembles *To. fici* with intact or cupulate conidiogenous cells that produce chains of rough-walled conidia (He et al. 2024c). *Torula yadongensis* has shorter (L/W ratio: 2 *vs*. 3.6) conidia than *To. fici*. The nucleotide differences for ITS, LSU, SSU, *tef*1, and *rpb*2 between *To. yadongensis* and *To. fici* were 10/489 (2%, without gaps), 21/780 (2.7%, without gaps), 5/991 (0.5%, without gaps), 18/798 (2.2%, without gaps), and 10/808 (1.2%, without gaps), respectively (Crous et al. 2015). Therefore, based on phylogenetic and morphological analyses, we propose a new species, *To. yadongensis*, from Yadong County, Xizang Autonomous Region, China.

## 4. Discussion

The Xizang Autonomous Region, China, is known for its extreme environmental conditions such as low temperatures, high UV radiation, and low oxygen levels (Chen et al. 2020; Wang et al. 2021). This provides a distinct habitat fostering the evolution of novel fungal taxa (Phurbu et al. 2024). In this study, we collected 19 specimens from the Xizang Autonomous Region, China, and identified a new genus, nine new species, and one new host record. Based on morphological characteristics and phylogenetic analyses, these taxa have been placed in two orders of Dothideomycetes: Astrosphaeriellaceae, Periconiaceae, Tetraplosphaeriaceae, Torulaceae, and one *incertae sedis* genus in Pleosporales (Zhang et al. 2012; Hongsanan et al. 2020), and Kirschsteiniotheliaceae in Kirschsteiniotheliales (Hernández-Restrepo et al. 2017).

*Triseptatospora* (Astrosphaeriellaceae, Pleosporales) was introduced by Konta et al. (2023). Species of Astrosphaeriellaceae were mainly found as sexual morph (Kuo and Goh 2019; Konta et al. 2023). *Triseptatospora yadongensis* has distinct synnemata, which is the key feature distinguishing it from other species in the Astrosphaeriellaceae (Liu et al. 2018; Wanasinghe et al. 2018; Dong et al. 2020; Crous et al. 2021; Luo et al. 2022; Konta et al. 2023). However, in the phylogenetic analysis, based on the genetic distance was not sufficient to provide strong evidence for the delineation of a new genus (Figure 4). Therefore, we retained our isolates in *Triseptatospora*, and for the first time, introduced a species from the Xizang Autonomous Region, China, into the genus (Konta et al. 2023), and proposed a new species, *T. yadongensis*. *Periconia* is the largest genus in Periconiaceae (Hyde et al. 2024b). Earlier members of this genus were placed in Massarinaceae. Based on phylogenetic analysis, Tanaka et al. (2015) suggested that Periconiaceae is a family distinct from Massarinaceae and Cai et al. (2024) first reported *Periconia* species in the Xizang Autonomous Region, China, based on morphology and phylogenetic analyses. In this study, we also introduce a new species and a new host record from the region. Tetraplosphaeriaceae was established by Tanaka et al. (2009) and consists of 10 genera (Zhang et al. 2023; Hyde et al. 2024b). In this study, based on phylogenetic and morphological analyses, we introduce a new genus, *Neotriplosphaeria*, to accommodate *N. yadongensis*, and a new species, *Te. linzhiensis*. *Torula,* is the most speciose genus in the Torulaceae with 542 epithets (He et al. 2024c). However, most species are not supported by molecular data (He et al. 2024c). Torulaceae currently has six genera introduced, namely, *Cylindrotorula*, *Dendryphion*, *Neopodoconis*, *Neotorula*, *Rutola*, and *Torula* (Qiu et al. 2022; Wang et al. 2023b). There is controversy over the division of some genera. Qiu et al. (2022) synonymised *Sporidesmioides* and *Rostriconidium* with *Neopodoconis*, Wang et al. (2023b) accepted *Neopodoconis* as a synonym of *Rostriconidium* and pointed out that there is insufficient evidence to synonymize *Sporidesmioides* with *Neopodoconis*. He et al. (2024c) proposed *Torula longiconidiophora* (Tian et al. 2023) as a synonym of *Torula sundara* (Jayawardena et al. 2022) based on phylogenetic and morphological analysis while investigating the relationships among species in the genus *Torula* (He et al. 2024c). Species of *Torula* are mainly distributed in Asia, Europe, North America (Jayawardena et al. 2022; Dong et al. 2023; He et al. 2024c). Both *To. dingjieensis* and *To. yadongensis* collected from Xizang Autonomous Region, China, add to the growing evidence that this region has a rich and largely unexplored fungal diversity. *Trichobotrys*, an *incertae sedis* genus, was placed in Pleosporales (Hyde et al. 2024b). To date, *Trichobotrys* includes four species supported by molecular data and morphological evidence, namely *Tr. effusus*, *Tr. meilingensis*, *Tr. sinensis*, and *Tr. yunjushanensis* (D’Souza and Bhat 2001; Phookamsak et al. 2024; Zhang et al. 2024). We constructed a phylogenetic tree (Figure 6) of *Trichobotrys* based on five genes (LSU, SSU, ITS, and *tef*1), and the results showed that *Trichobotrys* formed a similar topology to Phookamsak et al. (2024) and Zhang et al. (2024), with *Gregarithecium* having strong statistical support (100% ML/1.00 PP) in Dictyosporiaceae. Therefore, we propose the placement of *Trichobotrys* in Dictyosporiaceae, which further supports the conclusion proposed by Zhang et al. (2024). Kirschsteiniotheliales is a small order within Dothideomycetes (Hyde et al. 2024b). Species of *Kirschsteiniothelia* in China are primarily reported in the provinces of Guizhou, Hainan, Sichuan, and Yunnan (Tang et al. 2024; Xiao et al. 2025). In this study, we first report two species of *Kirschsteiniothelia* from Xizang Autonomous Region, China, including two new species *K. linzhiensis* and *K. yadongensis*.

The Xizang Autonomous Region, with its extreme environmental conditions, is a significant but underexplored area for fungal biodiversity. This study focused on microfungi from the Plateau, particularly within the orders Pleosporales and Kirschsteiniotheliales, both of which belong to the class Dothideomycetes. Fungal diversity plays a crucial role in the Xizang Autonomous Region by enhancing biodiversity understanding and ecosystem health (Hyde et al. 2024a; Phurbu et al. 2024; He et al. 2025). Xizang Autonomous Region, China, continues to be a rich source of fungal biodiversity. Further exploration of this unique environment is likely to uncover even more undiscovered fungal diversity.
